# State-level pathogen prevalence is associated with fewer interracial marriages and more racially restrictive dating preferences

**DOI:** 10.1093/pnasnexus/pgag356

**Published:** 2026-10-17

**Authors:** Jason D Freeman, Kodai Kusano, Joanna Schug

**Affiliations:** Department of Psychological Sciences, William & Mary, Williamsburg, VA 23187, USA; School of Psychology, University of Sussex, Falmer, Brighton BN1 9RH, United Kingdom; Department of Psychology, New York University Abu Dhabi, Saadiyat Island, Abu Dhabi, UAE; Department of Psychological Sciences, William & Mary, Williamsburg, VA 23187, USA

**Keywords:** pathogen prevalence, endogamy, mate selection, racial bias, interracial relationships

## Abstract

The behavioral immune system framework has linked higher levels of environmental pathogens with increased racial prejudice and xenophobia, suggesting that avoidance of racial outgroups may be an evolved strategy to avoid infection by pathogens and parasites. Although previous research has found a correlation between pathogen prevalence and racial bias on both regional and individual levels, few studies have examined whether this pattern predicts real-world outcomes. This paper presents two studies linking ecological pathogen prevalence to US state-level variation in interracial marriage (Study 1) and stated racial preferences on an online dating site (Study 2). States with higher pathogen levels tended to have lower-than-expected rates of interracial marriage, and online daters from these states were more likely to indicate more restrictive racial preferences. These results provide support for the pathogen prevalence hypothesis of prejudice and out-group bias by linking environmental pathogen levels to race-based preferences in marriage and dating.

Significance Statement This research contributes to our understanding of the complex interplay between environmental factors, psychological biases, and real-world social outcomes by examining the associations between ecological-level pathogen prevalence and US state-level rates of interracial marriages and racial preferences displayed on online dating profiles. The findings suggest that regions with higher rates of pathogen prevalence have lower rates of interracial marriage and a higher likelihood of individuals expressing racially restrictive dating preferences. These findings have implications for psychology and public health, underscoring the value of understanding how ecological factors impact human interpersonal behavior.

Although American attitudes toward interracial marriage have grown significantly more positive over the years, actual rates of interracial marriages remain relatively low (1–3). Many explanations for lower-than-expected rates of interracial marriage have been proposed. Some implicate psychological factors like prejudice and xenophobia, whereby people harbor negative attitudes toward individuals of certain ethnic backgrounds (4) and to interracial relationships in general (5–9). Others point to structural and geographic factors that restrict intergroup contact and the subsequent opportunities to form interracial relationships, such as racial clustering and segregation. Still, interracial marriages remain much rarer than would be expected given demographic factors alone (10).

Another perspective implicates psychological and structural explanations for low rates of interracial marriage, suggesting that ecological factors may drive the prevalence of psychological tendencies associated with xenophobic behavior. Research from the pathogen stress hypothesis argues that levels of environmental pathogens and parasites may drive regional variation in social behavior (11). This hypothesis, grounded in work on the “behavioral immune system” (BIS), suggests that social stigma may be an evolved response to avoid individuals who may harbor pathogens (12–15).

According to the BIS framework, infection by pathogens and parasites has been a risk of social contact since the first human societies and continues to be a threat today (16, 17). Just as non-human animals such as mice or lobsters have evolved mechanisms for avoiding compatriots infected by pathogens (18, 19), humans are proposed to likewise demonstrate behaviors tuned to avoiding infection by pathogens and parasites (13, 20). As unfamiliar others are more likely to harbor pathogens to which one does not have immunity (21), humans may have evolved psychological tendencies to avoid contact with strangers (22) or those who show phenotypic characteristics atypical of members of their own group (15). Given that external physical cues related to race and ancestry are often used as markers of group membership (23), activation of the BIS may result in xenophobic attitudes and behaviors that reduce rates of intergroup contact, resulting in fewer interracial marriages.

According to the pathogen stress hypothesis, xenophobic tendencies may be amplified in regions where people tend to be exposed to higher levels of pathogens and parasites. Consistent with this perspective, several studies linked pathogens and disease avoidance to xenophobic attitudes. On an ecological level, higher levels of pathogens have been linked to regional variation in psychological bias (11) and internet searches for racial slurs (24). On an individual level, several studies have linked disease avoidance to xenophobic attitudes (15), although laboratory studies examining the psychological impact of pathogens have produced inconsistent results (13, 25) or found inconsistent patterns (16) linking the BIS to xenophobia. Still, there is a dearth of research that has examined whether the prevalence of environmental pathogens predicts real-world outcomes subject to the influence of prejudice. One such outcome is the decision about whom to date or marry.

We address this lack of data on concrete real-world behavioral outcomes in the following studies by examining the link between pathogen prevalence and interracial marriage (Study 1) as well as stated racial preferences on online dating profiles (Study 2) in the United States. We expect that psychological mechanisms geared toward avoiding pathogens are highly relevant to interracial dating and marriage in high-pathogen regions. Indeed, psychological components of the BIS such as pathogen disgust are highly relevant in mating domains (26, 27) as engaging in sexual activity carries a high risk of infection by pathogens (28). Furthermore, psychological mechanisms geared toward avoiding pathogens have been shown to influence mate preferences in self-report studies (29, 30). However, to our knowledge, no research has examined whether regional variation in pathogen prevalence relates to racial bias in real-life mating domains with ecologically valid data. The present studies complement individual-level studies using experimental inductions of pathogen cues, which have yielded mixed results in previous studies (13, 25) by providing evidence from real-world social behavior.

Based on previous studies that have linked pathogens and the BIS with mating decisions and racial xenophobia, we expect US states characterized by high pathogen levels to have lower-than-expected rates of interracial marriage and more racially restricted dating preferences. In Study 1, we test this prediction with nationally representative data on interracial marriage in the United States. In Study 2, we test the same predictions by examining a large sample of online dating profiles in all 50 US states, assessing daters’ reported willingness to date someone outside of their racial group.

## Results

### Study 1

We conducted a series of regression models predicting rates of interracial marriage. First, we examined a baseline model predicting interracial marriage rates with the Diversity Index, which represents the probability that two people chosen at random from a given state would be from two different racial/ethnic groups. Consistent with prior work (2), demographics strongly predicted variation in interracial marriages, as indicated by a strong positive association between the Diversity Index and interracial marriages (*β* = 0.61, 95% CI [0.38, 0.84], *P* < 0.001), a negative association between the Diversity Index and intra-racial marriages between Whites (*β* = −0.71, 95% CI [−0.91, −0.50], *P* < 0.001) and a positive association for all other racial/ethnic groups (*β* = 0.67, 95% CI [0.46, 0.89], *P* < 0.001).

Adding pathogen prevalence to the models showed that pathogen prevalence was significantly associated with interracial marriages, *β* = −0.75, 95% CI [−1.00, −0.50], *P* < 0.001 (see Model 1 in Table 1), indicating that states with greater pathogen prevalence (e.g. Alabama, Louisiana, Figure 1) tended to exhibit lower-than-expected rates of interracial marriage after adjusting for the baseline rate of encountering racially different groups. Pathogen prevalence was also related to higher rates of intra-racial marriages among Whites *β* = 0.72, 95% CI [0.51, 0.92], *P* < 0.001 as well as intra-racial marriages for non-White racial/ethnic groups *β* = 0.59, 95% CI [0.30, 0.87], *P* < 0.001 (Models 2 and 3 in Table 1).^1^

**Figure 1:**
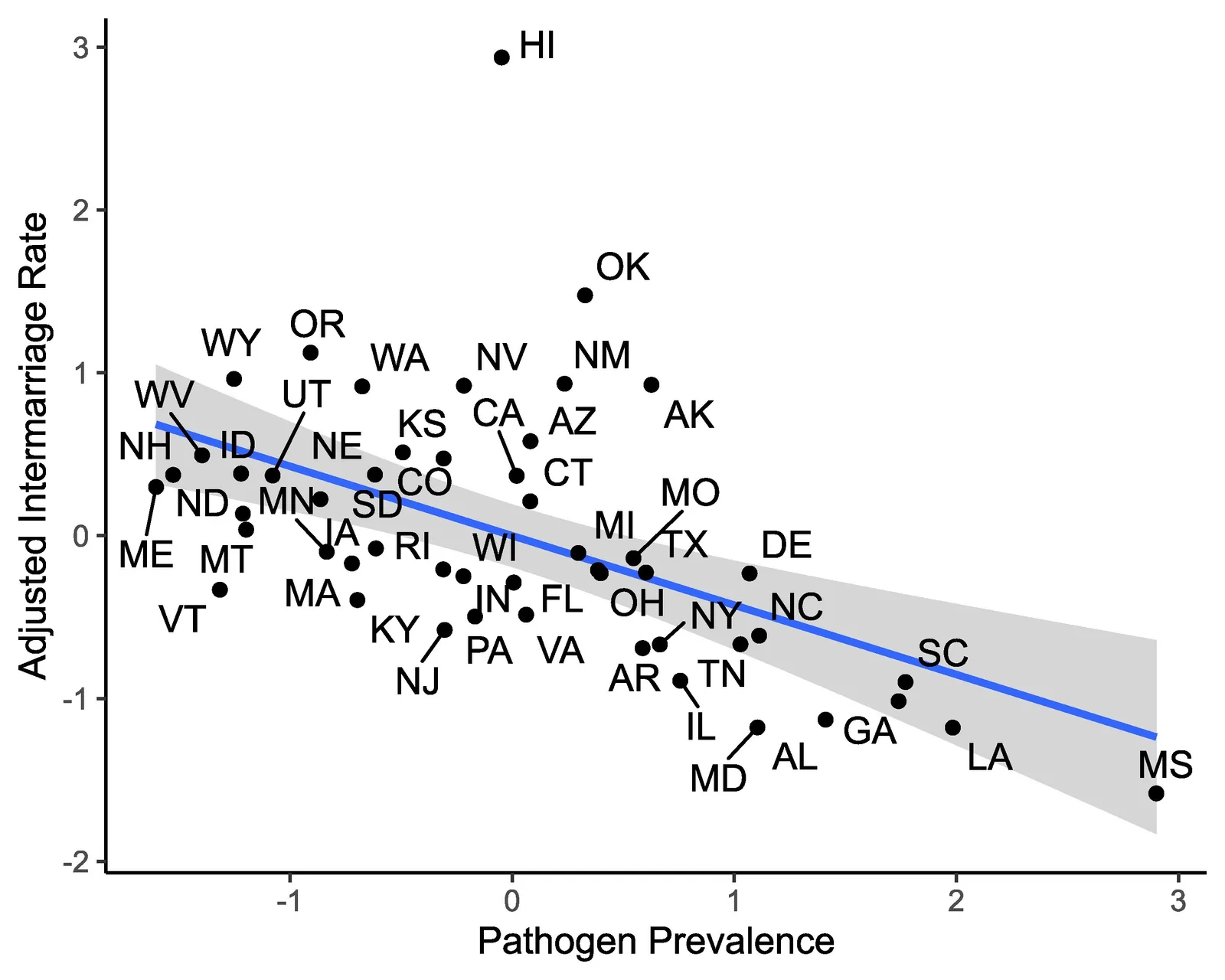
Association between state-level pathogen prevalence and interracial marriages in the United States.

**Table 1:** Regression results using state-level interracial and intra-racial marriage rates as the criterion variables.

	Model 1 Interracial marriages (all)		Model 2 Intra-racial marriages (White)		Model 3 Intra-racial marriages (non-White)	
Predictors	β	(95% CI)	β	(95% CI)	β	(95% CI)
Pathogen prevalence	−0.75***	(−1.00, −0.50)	0.72***	(0.51, 0.92)	0.59***	(0.30, 0.87)
Anti-miscegenation laws	−0.05	(−0.24, 0.14)	0.03	(−0.13, 0.19)	0.02	(−0.20, 0.24)
Population density	−0.28**	(−0.43, −0.12)	0.23***	(0.10, 0.36)	0.23*	(0.05, 0.40)
Diversity index	1.18***	(0.98, 1.39)	−1.25***	(−1.42, −1.07)	0.23^†^	(−0.01, 0.47)
*R* ^2^/*R*^2^ adjusted	0.76/0.74		0.84/0.82		0.68/0.66	

*Note. N* = 50(states). All coefficients are standardized. Anti-miscegenation laws represent a dummy variable of whether the state had anti-miscegenation laws at the point of the *Loving v. Virginia* ruling (no = 0; yes = 1). White intra-racial marriages are calculated as the ratio of marriages between two White partners to all marriages with at least one White partner. Non-White intra-racial marriages are calculated as the ratio of marriages between two people of the same race/ethnic background, excluding Whites, to the total number of marriages in which at least one partner is not White. ^†^*P* < 0.10, *P < 0.05, **P < 0.01, ***P < 0.001.

We also explored whether effects of pathogen prevalence remain after controlling for state-level racial bias and after accounting for potential geographic non-independence. The results, reported in the online supporting information, show that although state-level racial bias is highly predictive of interracial marriages, controlling for racial bias does not diminish the effect of pathogen prevalence. Rather, effects of state-level racial bias are largely explained by pathogen prevalence (Table S3).

These findings suggest that environmental pathogens may influence rates of interracial marriage across US states. States with higher levels of pathogens showed lower than expected rates of interracial marriage, after accounting for state-level diversity and other state-level control variables. The finding that state-level pathogen prevalence predicts decreased rates of interracial marriage adds another dimension to our understanding of how and why individuals choose to, or choose not to, date or marry outside their race.

One limitation associated with linking pathogen prevalence to interracial marriages is that who one marries is not simply a matter of personal preference—opportunity structures like racial endogamy can be induced by means other than the racial preferences of individuals themselves. Indeed, demographic factors were the largest predictor of state-level interracial marriages in the data presented above. Study 2 addresses this limitation by examining individuals’ *personal* racial preferences in dating partners that precede marriage decisions.

### Study 2

To test the hypothesis that higher state-level pathogen prevalence is associated with more restrictive racial preferences, we gathered data on stated racial preferences from online dating profiles in the United States. We examined correlations between pathogen prevalence and (i) the percentage of daters who preferred only their same race and (ii) the percentage of daters who endorsed a racial preference. We found significant correlations between state-level pathogen prevalence and the state-level tendency to list any racial preference on their profile, *r*(48) = 0.66, *P* < 0.001 and exclusively prefer their own race, *r*(48) = 0.46, *P* < 0.001 (Figure 2A and B).

**Figure 2:**
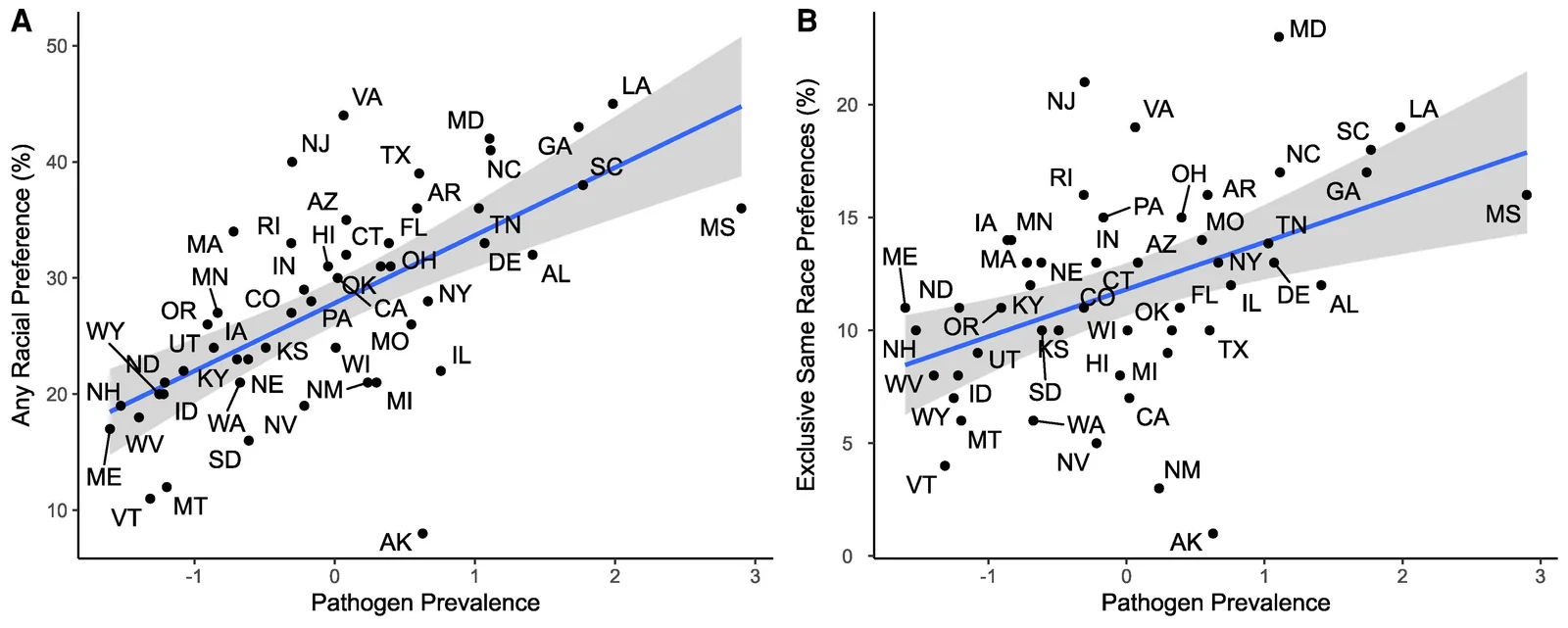
Association between state-level pathogen prevalence and reported racial preferences of online daters. A) The association between pathogen prevalence and the percentage of daters who included any racial restrictions on their profile. B) The association between pathogen prevalence and the percentage of users whose profiles indicated that they exclusively preferred their own race.

We used a multilevel model with daters nested within states using a logit link function to examine whether higher levels of pathogen prevalence would predict racial preferences in dating. This model examines the impact of pathogen prevalence, along with daters' race, gender, and education level, on the presence of any racial preference or a same-race preference on their dating profile (Table 2). Consistent with our hypotheses, daters in states with higher pathogen prevalence were more likely to exclusively prefer their own racial group (OR = 1.26, 95% CI [1.14, 1.39], *P* < 0.001) and indicate any restrictive racial preference (OR = 1.22, 95% CI [1.12, 1.32], *P* < 0.001). Compared to non-White daters, White daters showed more restrictive racial preferences (Same race only: OR = 2.91, 95% CI [2.55, 3.33], *P* < 0.001; any racial preference: OR = 1.18, 95% CI [1.09, 1.28], *P* < 0.001). Women were more likely than men to prefer someone of their own race (OR = 2.58, 95% CI [2.32, 2.87], *P* < 0.001) and indicate any racial preference (OR = 1.48, 95% CI [1.37, 1.59], *P* < 0.001).

**Table 2:** Results of models for all daters, with presence of exclusively same-race racial preferences and any racial preference or as the criterion variables.

	Same-race preference		Any racial preference	
Predictors	OR	(95% CI)	OR	(95% CI)
Fixed effects				
Pathogen prevalence	1.26***	(1.14, 1.39)	1.22***	(1.12, 1.32)
*Race* (*White vs. non-White*)	2.91***	(2.55, 3.33)	1.18***	(1.09, 1.28)
*Gender* (*woman*)	2.58***	(2.32, 2.87)	1.48***	(1.37, 1.59)
*Age*	0.99^†^	(0.98, 1.00)	1.01***	(1.01, 1.02)
*Education*	1.07	(0.96, 1.19)	1.09*	(1.02, 1.18)
Diversity index (capital city)	0.95	(0.87, 1.03)	1.08*	(1.01, 1.16)
Anti-miscegenation laws	1.45**	(1.17, 1.80)	1.47***	(1.24, 1.75)
Capital population density	1.28***	(1.17, 1.39)	1.22***	(1.14, 1.31)
(Intercept)	0.04***	(0.03, 0.05)	0.16***	(0.12, 0.20)
Random effects				
σ^2^_Residual_/τ_00 State_/ICC	3.29/0.05/0.01		3.29/0.03/0.01	
Marginal *R*^2^/conditional *R*^2^	0.141/0.153		0.055/0.065	

*Note*. OR, odds ratio. *N*(dating profiles) = 16,732, *N*(states) = 50. Individual level predictors are italicized. Race is coded as: 1 = White; 0 = non-White. Gender is coded as 1 = Woman; 0 = Man. Marginal *R*^2^ reflects variance explained by fixed effects only. Conditional *R*^2^ reflects variance explained by both fixed and random effects. ^†^*P* < 0.10, *P < 0.05, **P < 0.01, ***P < 0.001.

Next, we examined the effect of pathogen prevalence on racial preferences displayed by White daters and non-White daters separately (Table 3).^2^ Exploring whether the effects of pathogen prevalence occur both among White and non-White daters is important to elucidate potential mechanisms via which pathogens influence racial preferences. If the effect were to occur exclusively among White daters, this may suggest that greater pathogens increase existing pro-White racial biases. However, if the effect were found among both White and non-White daters, these results would suggest (as predicted by the hypothesis theory) that pathogen threat increases preferences for ingroup vs. outgroup mates.

**Table 3:** Results of random effects models presented separately for White and non-White daters, with exclusive same-race racial preference A) and the presence of any racial preference B) as the criterion variable.

A) Exclusive same-race preference				
	White daters		Non-White daters	
Predictors	OR	(95% CI)	OR	(95% CI)
Fixed effects				
Pathogen prevalence	1.25***	(1.12, 1.39)	1.24**	(1.06, 1.45)
*Gender* (*woman*)	2.51***	(2.23, 2.82)	3.07***	(2.34, 4.03)
*Age*	0.99*	(0.98, 1.00)	1.01	(0.99, 1.03)
*Education*	1.06	(0.94, 1.19)	1.15	(0.90, 1.46)
Diversity index (capital city)	0.94	(0.86, 1.04)	0.93	(0.83, 1.04)
Anti-miscegenation laws	1.49**	(1.18, 1.88)	1.40^†^	(0.98, 1.99)
Capital population density	1.32***	(1.20, 1.45)	1.08	(0.94, 1.24)
(Intercept)	0.12***	(0.08, 0.16)	0.02***	(0.01, 0.04)
Random effects				
σ^2^_Residual_/τ_00 State_/ICC	3.29/0.05/0.02		3.29/0.01/0.00	
Marginal *R*^2^/Conditional *R*^2^	0.105/0.118		0.124/0.126	

B) Any racial preference				
	White daters		Non-White daters	
Predictors	OR	(95% CI)	OR	(95% CI)
Fixed effects				
Pathogen prevalence	1.31***	(1.19, 1.43)	1.07	(0.95, 1.20)
*Gender* (*woman*)	1.36***	(1.25, 1.49)	1.90***	(1.66, 2.18)
*Age*	1.01	(1.00, 1.01)	1.02***	(1.01, 1.03)
*Education*	1.04	(0.96, 1.14)	1.23**	(1.07, 1.40)
Diversity index (capital city)	1.07	(0.98, 1.16)	1.00	(0.91, 1.10)
Anti-miscegenation laws	1.55***	(1.28, 1.88)	1.32*	(1.03, 1.69)
Capital population density	1.22***	(1.13, 1.32)	1.21***	(1.10, 1.34)
(Intercept)	0.23***	(0.18, 0.31)	0.10***	(0.07, 0.15)
Random effects				
σ^2^_Residual_/τ_00 State_/ICC	3.29/0.04/0.01		3.29/0.04/0.01	
Marginal *R*^2^/Conditional *R*^2^	0.064/0.076		0.053/0.064	

*Note*. OR, odds ratio. *N*(White dating profiles) = 12,061, *N*(Non-White dating profiles) = 4,671, N(states) = 50. Individual level predictors are italicized. Gender is coded as 1 = Woman; 0 = Man. Marginal *R*^2^ reflects variance explained by fixed effects only. Conditional *R*^2^ reflects variance explained by both fixed and random effects. ^†^*P* < 0.10, *P < 0.05, **P < 0.01, ***P < 0.001.

Results indicated that greater state-level pathogen prevalence positively predicted same-race preferences among both White and non-White daters. Results diverged for whether non-White daters listed any racial preference (i.e. the preference was not limited to their own race), for which pathogen prevalence was a significant predictor for White but not non-White daters.^3^ Similar results also emerged when using Bayesian hierarchical regression models with longitude and latitude included as Gaussian process spatial components to address concerns of geographic non-independence of adjacent states (Table S9). Additional analyses examining gender effects and the role of state-level racial biases are shown in the online supporting information.

## Discussion

These studies present ecologically valid data linking state-level pathogen prevalence with interracial marriages and stated racial preferences on an online dating platform. Study 1 showed that interracial marriage rates were lower than expected based on demographic availability in states with higher levels of pathogen prevalence. Likewise, Study 2 showed that daters in high-pathogen states were more likely to exclusively prefer their own race and indicate racial preferences. The significant effect of pathogen prevalence remained when accounting for individual-level factors (e.g. individual demographics), environmental factors (e.g. population density, state demographics, non-independence of states), and state-level racial bias (see analyses reported in online supporting information).

These findings extend ecological research linking regional pathogen prevalence with bias against racial out-groups (11) by demonstrating corresponding associations with consequential mate selection outcomes (30). These results should not be interpreted as evidence for or against effects of short-term manipulations of pathogen threat, which have produced inconsistent results. The present studies concern variation between regions and cannot identify individual or group-level processes responsible for that variation. Regional variation may emerge due to processes operating over longer time frames than those typically examined in studies using experimental manipulations of pathogen threat. Such processes may include transmission and reinforcement of social norms, developmental calibration to ecological conditions, or other evoked cultural responses to local disease ecologies. One potential pathway is biological immune activation, which has been linked to changes in automatic social behavior and intergroup evaluations in past research (32, 33). Further longitudinal and experimental research will be necessary to determine how regional pathogen prevalence becomes associated with restrictive preferences in dating and marriage.

The current studies are not without limitations. First, like much of the previous research examining the pathogen stress hypothesis, this work is correlational and cannot rule out alternative explanations. Future studies will be necessary to rule out potential confounds and unmeasured variables, ideally with other research designs. Other limitations of this work include the representativeness of the online dating sample. While online dating has grown more popular, only 30% of adults in the United States have used an online dating platform, and users of online dating sites tend to be relatively younger and more educated than non-users (34). Future research should extend upon these findings using representative samples of individuals who are seeking a romantic partner both online and off, and generalize beyond Western, Educated, Industrialized, Rich, and Democratic populations (35).

Furthermore, racial preferences displayed on dating profiles may not directly correspond to underlying racial preferences. For instance, in regions with low racial diversity there may be little need for majority group members to restrict their racial preferences to the ingroup, as there would be little chance of encountering those from statistical minority groups. Likewise, for individuals who belong to minority groups and for individuals in regions with fewer potential partners in general (e.g. low relational mobility (36)), limiting one's preferences to only members of one's own group would severely limit the pool of potential dating partners. Thus, the degree to which stated racial preferences reflect actual racial preferences may differ across racial groups and regions.

Finally, while these studies suggest that people in regions with higher levels of environmental pathogens tend to show more restrictive racial preferences in marriage and dating, future studies should examine proximate causal mechanisms for this tendency. One potential mechanism may be that increases in pathogens in an environment increase racial bias, which then leads to own-race preferences or other-race exclusion. However, although increases in state-level racial bias predicted fewer interracial marriages and more restrictive dating preferences, the effects of pathogen prevalence remained when controlling for state-level racial bias in both studies. We report results related to racial bias and additional mediation models in the online supporting information showing that effects of state-level racial bias are largely mediated by pathogen prevalence. As examining mediation effects is challenging with regional-level analyses such as reported in this study, future studies should examine within-person or longitudinal effects (such as the impact of the COVID-19 pandemic (37)) or use more granular regional data that allow for more rigorous tests of causal and mediation mechanisms.

Overall, these findings have implications across disparate fields of study, including social psychology and public health. If future causal research confirms that pathogen prevalence contributes to these outcomes, public health interventions reducing disease burdens could potentially have broader downstream effects on intergroup relations. These results are particularly important given that interracial marriage has been shown to be a reliable indicator of decreasing social distance between racial groups in the United States (38). As stigma is still experienced by many interracial couples (39), broadening our understanding of the ecological antecedents of such stigmatization is critically useful, particularly as we consider actions which may help alleviate it.

## Materials and methods

### Study 1

To examine whether high levels of pathogen prevalence on a regional level correspond to lower-than-expected rates of interracial marriage in the United States, we compiled data from several sources.

#### Outcome variable: interracial marriage rates

We followed the method used by the Pew Research Center (1) to index rates of interracial marriage across all 50 US states, based on a 3-year (2008–2010) combined sample of the American Community Survey (ACS) data. As spousal records are linked in the ACS data, they constructed a dataset consisting of demographic information for linked pairs of newly married individuals (those married within the “past 12 months” whose marital status was coded as “married, spouse present”). Couples in this dataset were then subdivided by racial group, with individuals identifying as being of non-Hispanic origin divided into the categories of White, Black, Asian and Pacific Islander, American Indian and Alaska Native, two or more races, or a residual category (“Some other race”). A “married out” percentage was then calculated for each state by dividing the number of interracial couples by the total number of married couples in that state. As this study did not report rates of intra-racial vs. interracial marriage separately for different ethnic groups, we accessed the original ACS data and computed the rate of intra-racial marriages for Whites (White intra-racial marriages/total marriages involving at least one White spouse) and non-Whites (total number of marriages between two non-White people of the same racial background/total number of marriages involving at least one non-White spouse).

#### Expected rates of interracial marriage

Racial demographics play a critical role in determining the baseline prevalence of interracial marriage (3, 40). Based on racial demographics alone, interracial couples should be expected to be 11 times more likely in Hawaii, the most ethnically diverse US state, compared with Vermont, where non-Hispanic Whites make up over 90% of the population. To accurately estimate how pathogen prevalence influences interracial marriage when opportunities to marry outside one's racial group are equal, it is necessary to control for state-level demographic differences. We do so by adjusting for the Diversity Index calculated from 2010 census data, which captures the probability that two people meeting at random will be from two different racial or ethnic backgrounds (41).

#### Pathogen prevalence

We use scores from Fincher and Thornhill's Parasite Stress USA index (14) of reported cases of infectious diseases (both zoonotic and non-zoonotic) tracked by the Centers for Disease Control and Prevention's annual *Morbidity and Mortality Weekly Report*'s “Summary of Notifiable Diseases, United States” from the years 1993 to 2007 across all 50 US states. This analysis resulted in a population-adjusted average z-score across these 15 years for each state.

#### State-level control variables

Additional models accounted for other factors that contribute to differences in interracial marriages. As states with historically longer-lasting anti-miscegenation laws have been shown to also have lower rates of interracial marriage (2), a dummy variable was created coding for whether states had anti-miscegenation laws in place at the time of the *Loving v. Virginia* Supreme Court decision (“1”) or not (“0”). This variable also takes into account non-independence of states arising from geographical clustering, as it closely mirrors the geographical distinction between southern and northern states.^4^ We also controlled for population density, since a higher density has been theorized to drive increases in interracial contact (9). As previous work has shown that pathogen prevalence is highly correlated with racial bias in the United States (11), we also examined effects of state-level racial bias using a measure of implicit bias computed by O'Shea and colleagues (11) using a large body of Implicit Association Task data from Project Implicit (42) and present the results in online supporting information.

### Study 2

Study 2 uses data from online dating profiles to more precisely measure the influence of pathogen prevalence on individual racial preferences in dating. While past research has examined racial preferences on online dating profiles (43, 44), and linked racial preferences in online dating profiles to environmental factors like population diversity (45), it is unknown whether environmental pathogens may help shape stated racial preferences. Based on the pathogen stress hypothesis, we predicted that individuals living in states with higher historical pathogen prevalence would be more likely to endorse specific racial preferences on their online dating profiles that exclude racial out-groups (e.g. preferring only daters of one's same race).

#### Sample

We examined a sample of online dating profiles displayed on a popular online dating platform in the United States. This platform was chosen for its unique layout, which (at the time of data collection) displayed daters’ stated racial preferences. We followed guidelines (46) to determine our ideal sample size (although the level-2 sample size was fixed at 50 US states), we sought to gather information posted on the profiles of 200 men and 200 women within each of the 50 US states to detect a small or medium effect with a target power of 0.80. All procedures were reviewed and approved by the Protection of Human Subjects Committee at William & Mary (Protocol Title: Pathogen Prevalence as a Predictor of Racial Preferences in Dating). The approved IRB application explicitly specified that individual informed consent would not be obtained because the study involved analysis of existing information available on an online dating platform. No personally identifying information, including names or photographs (i.e. profile pictures), was stored or analyzed, and no individuals were contacted or subjected to experimental procedures.

We compiled a dataset including information from the profiles of the 200 most recently active heterosexual men and women (if available) between the ages of 18 and 45 who live within 10 miles of the most populous zip code in each state capital city. We chose state capital cities because they are typically centrally located in their states, thereby avoiding spillover from adjacent states. Capital cities are also associated with only a modest increase in urbanization (47) and thus better represent state populations than major metropolitan areas such as New York City, Los Angeles, or Chicago.

For each profile, we collected text data identifying the racial identity of the dater and any racial preferences they indicated for a potential partner as well as their age, gender, and education level. A total of 170 blank (no information provided) or duplicate (i.e. profiles that had identical “handle” names, age, location, and partner age/location preference) were removed from the dataset. Daters whose profiles did not indicate their own race were removed from our final dataset (a total of 519 profiles). The final dataset consisted of 16,732 dating profiles (8,041 women and 8,691 men), with an average of 334.64 profiles analyzed per state. The mean age of our sample was 33.7 (*SD* = 6.3), 72.1% of daters identified as White and 40.5% of profiles indicated that the user had a college degree (Table S1).

#### Outcome variables

To determine the potential influence of pathogen prevalence on racial preferences, we created a binary outcome variable indicating whether or not a dater listed a racial preference on their dating profile (0 = did not, 1 = did). It should be noted that what constitutes a racial preference in this dataset is quite broad. For example, a dater may choose not to list any racial preference, list only their same race, or any combination of two or more races, even to the point of including all but one or a few racial categories. Thus, we created another outcome variable, indexed as same-race preference, indicating whether the dater endorsed only their same race on their preferences list (0 = did not exclusively prefer own race, 1 = exclusively preferred own race).

#### Control variables

As in Study 1, we included a dummy variable representing whether states had anti-miscegenation laws in place at the time of the *Loving v. Virginia* Supreme Court decision (1) or not (0). Additional dummy variables for a participant's education level (0 = less than associate degree, 1 = associate degree or above), gender (0 = man, 1 = woman), and race (0 = non-White, 1 = White) were included. To control for the racial diversity within each capital city, we again computed the Diversity Index using 2020 census data from each city to reflect the likelihood that two people chosen at random from the city population would be of two different racial groups.

## Supplementary Material

pgag356_Supplementary_Data

## Data Availability

Data and analysis scripts can be found at https://osf.io/mb76w.

## Footnotes

1 Results also emerged when using Bayesian hierarchical regression models with longitude and latitude included as Gaussian process spatial components (31) to address concerns of geographic nonindependence of adjacent states (Table S8).

2 Given uneven distributions of racial demographics across states, disaggregating data into discrete racial categories yields too few observations in some state to allow for state-level analysis. See Table S1 for a breakdown of the sample by demographic variables.

3 We present further analyses in Table S11 showing that these effects are driven primarily by non-zoonotic pathogens and not by zoonotic infections. In the source classification, the non-zoonotic index includes human-specific and multihost diseases capable of human-to-human transmission, whereas zoonotic diseases are transmitted to humans from nonhuman animal hosts and are not transmitted between humans. The associations with dating preferences were primarily observed for non-zoonotic disease prevalence. In particular, non-zoonotic disease prevalence significantly predicted the endorsement of a racial preference among non-White daters, although the corresponding association using the combined pathogen-prevalence measure reported in the main analysis was not significant.

4 The presence of anti-miscegenation laws at the point of the *Loving v. Virginia* is highly correlated with whether a state is part of the US South. Only one Southern state (Maryland) repealed anti-miscegenation laws prior to the *Loving v. Virginia* decision, and one non-Southern state (Missouri) had anti-miscegenation in place at the time of the *Loving v. Virginia* decision. Controlling for North vs. South region in lieu of anti-miscegenation laws produces nearly identical results. This implies that the negative effect of pathogen prevalence on interracial marriage rates is similar across North vs. South geographical regions. Additional models using Bayesian hierarchical regression model with Gaussian process spatial components (31) to address concerns of geographic non-independence are presented in the online supplementary information.
