# Supplementary material for: State-level pathogen prevalence is associated with fewer interracial marriages and more racially restrictive dating preferences
Source: PNAS Nexus. 2026 Oct 17;5(10):pgag356. doi: 10.1093/pnasnexus/pgag356 (PMC13712138; doi:10.1093/pnasnexus/pgag356)

PNAS nexus

Supplementary Information for

State-level pathogen prevalence is associated with fewer interracial marriages and more racially restrictive dating preferences

Jason D. Freeman[1,2], Kodai Kusano[3], & Joanna Schug[1]

[1]William & Mary, Department of Psychological Sciences, William & Mary, Williamsburg, VA, USA
[2]University of Sussex, School of Psychology, Falmer, Brighton, United Kingdom
[3]New York University Abu Dhabi, Department of Psychology, Abu Dhabi, UAE

Corresponding author: Joanna Schug, jschug@wm.edu

This PDF file includes:

Supporting text
SI References
Tables S1 to S26
Figures S1 to S3

Data and analysis scripts can be found at: https://osf.io/mb76w/

Demographic breakdown of sample

Demographics of the online dating profiles examined in Study 2 are presented in Table S1. Figure S1 presents heat maps of dating preferences and Figure S2 presents heat maps of interracial marriages and pathogen prevalence across U.S. States. Correlations among key measures are presented in Table S2.

The role of State-level Racial Bias

Study 1. As previous research has linked state-level pathogen prevalence with aggregated regional variation in implicit racial bias (O'Shea et al., 2020), we examined whether state-level implicit bias scores predicted rates of interracial marriages to determine if the effects of pathogen prevalence hold when controlling for state-level racial bias. We report additional mediation models for state-level racial bias in the "Exploration of potential mediators and mechanisms" section below.

For this study, we used state-level implicit bias "D-scores" compiled by O'Shea and colleagues (2020) based on data from Project Implicit (Xu et al., 2014) years 2015 to 2019 for White participants. Here, a D-score represents the average of the difference in mean reaction time to bias-congruent stimuli pairings (White-positive, Black-negative), subtracted from the mean reaction time for bias-incongruent stimuli pairings (White-negative, Black-positive), divided by the combined standard deviation of all trials in all blocks. We assume that aggregated implicit association test (IAT) scores represent the contextual strength of concept accessibility of racial bias across states, with a higher score indicating a greater prevalence of racial bias in a region (Payne et al., 2017; Payne & Hannay, 2021).

Results are presented in Table S3. Following the models presented in the main manuscript (Model 1), we examine two additional models. Model 2 includes state-level racial bias, and Model 3 includes both pathogen prevalence and state-level racial bias. As with pathogen prevalence, states with higher levels of racial bias showed lower levels of interracial marriages (β = -0.60, p<.001). Including both pathogen prevalence and state-level racial bias in the model (Model 3) showed that pathogen prevalence remained a stronger predictor of interracial marriages β = -0.58, p <.001, while the effect of racial bias was not statistically significant (β = -0.27, p=.058).

These results suggest that the effect of pathogen prevalence on decreased interracial marriages, while also predicted by state level racial bias scores, remained even when controlling for racial bias. However, these results must be interpreted with caution as racial bias scores reflect the attitudes of White participants toward White vs. Black stimuli, while the interracial marriage data reflect marriages across all racial categories. We present further analyses examining the role of racial bias on dating preferences shown by White daters toward Black individuals below.

Study 2. To examine whether the potential impact of pathogen prevalence on racial preferences in dating could be explained by racial bias on a psychological level, we compiled US state-level implicit bias scores through a large sample of race Implicit Association Test (IAT) data available via Project Implicit (Xu et al., 2014), for the five year period from 2015 to 2019. State-level scores were calculated using the average D-score for White participants in each state (Greenwald et al., 2003).

Given that the IAT data compare differences in reactions to White vs. Black target stimuli, we focus solely on White daters. We also include a model specifically examining White daters' tendencies to

exclude Black daters by creating a variable to indicate whether participants chose to exclude Black daters from their preferences (0 = did not exclude, 1 = excluded Black daters). Results are shown in Table S4. We found that state-level racial bias, as measured by state-level pro-White IAT scores, predicted both the existence of racial preferences and exclusive own-race preferences. Surprisingly, we did not find a significant effect of racial bias on exclusion of Black daters by White daters, after including pathogen prevalence in the same model (Table S4, Model C). We report additional mediation models exploring whether state-level racial bias can explain the relation between pathogen prevalence and racial preferences and interracial marriages in the section "Exploration of potential mediators and mechanisms" below.

Exploration of potential mediators and mechanisms

Although we show that pathogen prevalence predicts variation in both interracial marriages and racial preferences listed on online dating profiles, the mechanisms linking regional pathogens to racial preferences remain unknown. Previous studies have proposed that pathogen stress may influence a large suite of behaviors and psychological mechanisms, generally encouraging individuals to interact with ingroups vs. outgroups (Fincher & Thornhill, 2012) and increase tendencies that encourage social cohesion such as tighter social norms (Harrington & Gelfand, 2014), increased conformity (Murray et al., 2011), cooperation with ingroups (Imada & Mifune, 2021), and decreased relational mobility (Thomson et al., 2018). Examining whether these contextual factors might mediate the link between pathogen stress and interracial dating and marriage outcomes can help us understand how pathogen stress affects decision making in dating and mating contexts.

In order to explore potential mechanisms via which pathogens may impact interracial marriage and dating preferences, we conducted a series of causal mediation models using the mediation package in R (Tingley et al., 2014) to examine if pre-existing US state-level measures of cultural Tightness (Harrington & Gelfand, 2014), Collectivism (Vandello & Cohen, 1999), and the Strength of Family Ties (Fincher & Thornhill, 2012) mediate the relationship between pathogen prevalence and dating preferences or marriage decisions. We should note that because these analyses are on a state level (n=50) and correlational in nature, drawing definitive conclusions about causation based on these models is not possible. Rather, these models may help to guide future research that seeks to elucidate the mechanisms via which pathogens impact behaviors.

Following previous research (Horita & Takezawa, 2018), we created a state-level measure of conformity using data from the two most recent waves of the World Values Survey, in which state of residence for respondents was included, and calculated the percentage of participants who answered "obedience" when shown a list of traits (e.g., religious faith, obedience, unselfishness) and were asked which were important to teach children. As described above, we created a state-level measure of implicit bias using data from Project Implicit (Xu et al., 2014), for years 2015 – 2019 for White participants. We used measures of tightness-looseness (Harrington & Gelfand, 2014), collectivism (Vandello & Cohen, 1999), and strength of family ties (Fincher & Thornhill, 2012) reported in prior research. The results of these causal mediation analyses present a complex picture of how state-level cultural variables mediate the relationship between pathogen prevalence and dating preferences or marriage outcomes. Across nearly all models, pathogen prevalence has a significant average direct effect (ADE) on outcomes, indicating the existence of a robust direct relationship. Indirect mediating pathways, however, varied considerably depending on the cultural variable or specific outcome being measured.

Interracial marriage. When predicting state-level marriage rates (Table S5), the role of mediators was limited. State-level racial bias was the most notable mediator, significantly explaining part of the relationship between pathogen prevalence and both fewer interracial marriages (ACME = -0.17, $p < .05$) and more White intra-racial marriages (ACME = 0.013, $p < .05$). Cultural tightness also emerged as a significant mediator, but only for non-White intra-racial marriages (ACME = 0.15, $p < .05$). This may reflect the tendency for minority populations to encourage tighter social norms (Weaving & Gelfand, 2024). In contrast, collectivism, conformity, and family ties did not significantly mediate the link between pathogen prevalence and any of the marriage outcomes.

Dating Preferences. The analysis of individual-level dating preferences (Table S6) revealed more prominent involvement of cultural mediators. State-level collectivism was the strongest and most consistent mediator, significantly accounting for the association of pathogen prevalence with both expressing any racial preference (ACME = 0.0092, $p < .001$) and an exclusive same-race preference (ACME = 0.0069, $p < .001$), explaining 22% and 26% of the total effects, respectively. Other variables showed more specific mediating effects: state-level racial bias significantly mediated the association with same-race preferences (ACME = 0.0050, $p < .01$) but not for expressing any preference, whereas strength of family ties significantly mediated the association with expressing any racial preference (ACME = 0.0068, $p < .001$) and showed only a marginal effect for same-race preferences. Cultural tightness showed only a marginal mediating effect for same-race preferences (ACME = 0.0020, $p < .10$), and conformity was not a significant mediator in any model.

Shared variance in the relation between pathogens and state-level racial bias. We also examined the extent to which state-level pathogen prevalence accounts for overlapping variance in the relationship between state-level implicit bias and individual dating preferences (Table S7). We emphasize that these analyses do not imply a causal pathway (e.g., that racial bias influences pathogen prevalence), but rather highlight the degree of statistical overlap and the relative robustness of pathogen prevalence as a predictor. Among all daters, pathogen prevalence fully accounted for variation in the effect of implicit bias on having any racial preference (ACME = 0.0129, $p < .001$; Percent Accounted for = approximately 100%). White daters showed similar patterns, with 84.6% of the total effect accounted for (ACME = 0.0156, $p < .001$), while no significant mediation was found for non-White daters. For same-race preferences, indirect effects were also significant across all groups, though less pronounced, with 36.8% of the total effect accounted for among all daters and 30.0% among White daters. Among non-White daters, the estimated proportion mediated was 58.9%, although neither the total effect nor the proportion mediated was statistically significant in this subgroup. Notably, White daters also showed strong mediation for the exclusion of Black partners (ACME = 0.0145, $p < .001$; Percent Accounted for = 74.6%). Overall, these analyses indicate substantial statistical overlap between pathogen prevalence and state-level racial bias in predicting racial dating preferences, particularly among White daters.

Geographical non-independence

One concern that has been raised in prior ecological-level research (such as across nations, cultures, and geographic regions) is that the geographic regions are not independent. Neighboring regions such as states and countries may share many similarities in culture and ecology, and breaking these underlying dimensions into multiple independent data points can inflate the potential for false-positive results. In the case of the current research, pathogens are not evenly distributed across regions. In the United States, southeastern states tend to rank higher on measures of

disease prevalence. This has been attributed to factors including temperature (which is geographically distributed), as warmer and more humid climates tend to be hospitable to certain types of pathogens and parasites (Mathison & Pritt, 2023). Similarly, states may have shared immigration and migration patterns and cultural history, such as anti-miscegenation laws that historically criminalized interracial marriages. Several methods have been proposed to reduce the probability of false positives resulting from non-independence, which we will discuss below.

Regional variables as controls. One method for addressing non-independence in state-level data is to control for geographical regions using regional dummy variables. Including a regional dummy allows us to test whether observed effects remain consistent when comparing the southern and northern United States. To control for regional differences, our main analyses reported in the main manuscript included a dummy variable indicating whether a state had anti-miscegenation laws at the time of the Loving v. Virginia decision—a variable strongly correlated with Southern regional membership. Nearly all Southern states retained these laws until the Loving v. Virginia decision (except Maryland, which repealed the laws earlier), while Missouri was the sole non-Southern state with such laws in place. This means that the variable itself is geographically clustered, and including it as a predictor addresses the non-independence problem to some degree (Akaliyski, 2024). Replacing the anti-miscegenation dummy variable with a North versus South dummy variable produces nearly identical results, suggesting that the negative association between pathogen prevalence and interracial marriage rates is similar across northern and southern regions.

Three-level models. Another approach to reducing false positives arising from geographical non-independence is to account for interdependence by including random intercepts for higher-level units, such as regions or divisions, in a multilevel regression (Kuppens & Pollet, 2014). This method has been successfully applied with ecological data at the country and U.S. state levels (Kusano & Kemmelmeier, 2018; 2020). Such models typically show that although regional interdependence can be substantial, including random intercepts for broader regions affect the observed effects only trivially. This approach did not change any of the conclusions made in the main manuscript.

Bayesian random effects models controlling for longitude and latitude. Beyond simply controlling for fixed effects, which may differ between regional units, recent statistical advances have allowed for more precise control of spatial non-independence. Bayesian random effects regressions can be explicitly fitted with geographic information to model spatial non-independence by allowing regions to covary depending on their geographic distance through inclusion of a Gaussian process. A recent modelling study examining different methodologies for addressing spatial non-independence (Claessens et al., 2023) found that Bayesian spatial Gaussian regression models significantly reduced false positives compared with traditional methods such as inclusion of fixed geographic control variables, while retaining high statistical power to detect true effects.

We employed a series of Bayesian hierarchical regression models with a Gaussian process spatial component to examine the relationship between outcome variables (any racial preferences, exclusive same-race preferences) and multiple predictors while accounting for state-level variation and spatial autocorrelation. The results of the models for Study 1 are presented in Table S8, and the results for Study 2 are presented in Table S9. The estimated spatial parameters include spatial standard deviation (sdgp) and length-scale (lscale) indicating moderate spatial variation. Regression coefficients were estimated using Markov Chain Monte Carlo (MCMC) sampling via the No-U-Turn Sampler (NUTS). The Rhat values (~1.00) indicate model convergence, while the effective sample sizes (Bulk ESS and Tail ESS) suggest reliable parameter estimates. Models for

Study 2 include a random intercept for state, allowing baseline differences across states and a Gaussian process spatial component, capturing spatial dependencies in the data. These analyses find that the effect of pathogen prevalence remains even in models that take geographical non-independence of observations into account.

Comparing zoonotic vs. non-zoonotic Pathogens

We also examined whether associations between pathogen prevalence and stated racial preferences differ according to pathogen transmission mode. Following the classification used by Fincher & Thornhill (2012), the non-zoonotic disease index includes human-specific and multihost diseases capable of human-to-human transmission, whereas zoonotic diseases are transmitted to humans from nonhuman animal hosts and, under this classification, are not transmitted between humans. The parasite-stress framework predicts that non-zoonotic diseases should be more closely associated with human social behavior because their transmission can potentially be reduced by avoiding contact with other humans.

Results of Study 1 and 2 for zoonotic and non-zoonotic infections are shown in Table S10 and S11, respectively. Non-zoonotic disease prevalence significantly predicted both the endorsement of any racial preference and an exclusive same-race preference among all daters, White daters, and non-White daters. By contrast, zoonotic infections do not generally impact stated racial preferences. Notably, the association between non-zoonotic disease prevalence and the endorsement of any racial preference among non-White daters is significant, although the combined measure of pathogen prevalence (including both zoonotic and non-zoonotic infections) presented in the main manuscript was not significant. These analyses are consistent with regional non-independence, in the sense that both Zoonotic and non-Zoonotic parasites and infectious diseases tend to flourish in warmer and more humid climates such as in the US South and Southeast. However, separating zoonotic and non-zoonotic diseases does not itself account for spatial non-independence; that issue is addressed more directly by the spatial models reported elsewhere in the Supplementary Material.

Exploration of Gender Effects

Previous research suggests that, like immune function in general (Bouman et al., 2005), mechanisms associated with the BIS tend to be more pronounced for women than men. For instance, women have also been shown to score higher in disgust sensitivity, particularly in sexual domains (Al-Shawaf et al., 2017; Tybur et al., 2011). One cross-cultural study of 48 nations (Schmitt, 2005) found that increased prevalence of non-zoonotic disease was associated with more restricted sociosexuality among women, but not in men. In addition to influencing sexual behavior differentially, pathogen prevalence has been shown to influence women's preferences for facial masculinity (DeBruine et al., 2010) and physical attractiveness (Gangestad & Buss, 1993). This effect has been examined in laboratory experiments, wherein women primed with pathogen threat have been shown to alter their mate preferences (Lee & Zietsch, 2011).

We examined Gender × Pathogen Prevalence interactions to determine whether the effect of pathogen prevalence on dating preference differs by gender (Table S12). First, women on average were much more likely to both exclusively prefer their own race and indicate any racial preference. The pathogen prevalence × gender interaction term was significant for the presence of any racial preference, but not for exclusive same-race preferences. The significant interaction between gender and pathogen prevalence for the presence of any racial preference reflected that, while men generally had lower baseline odds of indicating a racial preference compared to women, men

exhibited a steeper slope, causing the gender difference to diminish in states with higher pathogen prevalence (see Figure S3).

Alternative Hypotheses

1. Life History Theory

Prior work has noted that common state-level measures of pathogen prevalence are highly correlated with the percentage of a state's population that is Black/African-American (Hackman & Hruschka, 2013). Within some applications of Life History Theory (LHT), race (particularly being Black in the United States) has served as a proxy for high extrinsic risk, given longstanding inequalities in exposure to poverty, violence, or instability. From this perspective, one alternative explanation is that patterns explained using Parasite Stress Theory (PST) may instead reflect greater preference for "fast" life history strategies in areas with relatively larger Black populations.

At the same time, using the percentage of a given state that is Black in this way raises conceptual limitations. As a highly coarse, socially constructed variable, it reflects structural racism, segregation, SES, and regional history rather than a single, clearly defined mechanism of risk. For this reason, racial composition may be an imprecise proxy of extrinsic risk. By contrast, pathogen prevalence is a direct ecological risk indicator and therefore may be a more appropriate extrinsic risk factor. In addition, racial restrictiveness inherently limits the pool of potential partners and may be more consistent with a cautious, "slow"-life history mating strategy, contrary to the "fast"-strategy behaviors predicted by life-history theory (Moon et al., 2018, 2019). More broadly, the two frameworks operate at different points in a possible causal chain: LHT generally treats outcomes such as sexually transmitted disease rates and earlier reproduction as consequences of fast strategies in harsh ecologies, whereas PST accounts treat pathogen threats as a factor that increases in-group preference and social conservatism. These perspectives are therefore not necessarily mutually exclusive.

To evaluate the LHT account more directly, we examined additional models incorporating common state-level variables associated with "fast" life history strategies including state-level Teen Pregnancy Rate (Table S13 and S14) and Age at First Birth (Table S15 and S16). Across both Study 1 and Study 2, these life-history variables did not significantly predict intermarriage or dating preferences when entered with pathogen prevalence (Table S17-S20). When entered simultaneously, pathogen prevalence remained significant while the life-history variables did not. Taken together, this pattern suggests the present findings are not readily explained by state-level variation in fast life-history profiles alone.

2. Proportion of Black residents

A related question is whether the proportion of Black residents in a state offers a more substantive explanation than pathogen prevalence. One possibility is that people in states with larger Black populations might show higher levels of racial bias more generally. To address this, we examined models including state-level implicit racial bias (IAT scores) as a covariate. In those analyses, state-level pathogen prevalence remained a robust predictor of the focal outcomes (Tables S3 - S4).

A second possibility is that people in states with larger Black populations may be especially likely to exclude Black daters from their dating preferences, which could make the observed effects appear to reflect broader racial exclusion when they are in fact driven by avoidance of one particular group. Several of our analyses are inconsistent with this interpretation. Models stratified by racial group

(Table 3; Bayesian spatial models in Table S9) show that pathogen prevalence predicts greater preference for same-race partners among both White and non-White daters and intra-racial marriage among non-Whites. This pattern is more consistent with a generalized in-group preference, not a pattern driven specifically by avoidance of Black partners.

We completed several additional analyses using the Study 2 data to test whether the association between pathogen prevalence and racial preferences reflects general in-group preference rather than targeted avoidance of any single group. Across these models, higher pathogen prevalence predicts:

1) A greater number of races excluded from a dater's preferences (Table S21);
2) An increased likelihood of excluding 2 or more races (Table S22);
3) The exclusion of other non-Black groups (e.g., Asian or Hispanic daters) (Table S23);
4) Specific exclusion of individual racial categories by daters not of that same group (e.g., exclusion of Asian daters by non-Asian daters, White daters by non-White daters, etc.) (Table S24).

Overall, these findings are consistent with the hypothesis that pathogen stress is associated with broader out-group avoidance or stronger in-group preference, rather than a targeted aversion toward any one specific racial category.

3. Examining the Role of STIs vs. other pathogens

Another issue concerns whether associations attributed to parasite stress may be driven primarily by sexually transmitted infection (STI) rates, which some life history accounts link to "fast" strategies and promiscuity (Hackman & Hruschka, 2013). From the perspective of PST, however, STIs are not thought to be a special case, but rather one form of human-specific, non-zoonotic infectious disease (Brown et al., 2024). Pathogens transmitted via sexual contact are also theoretically highly relevant in mate selection contexts, as engaging in sexual activity carries a high infection risk (Al-Shawaf et al., 2017). Accordingly, an association between STI prevalence and interracial dating behavior is not inconsistent with the broader Parasite-Stress interpretation.

We nevertheless examined this issue directly using additional models that replaced the Parasite Stress USA measure with a measure of pathogen-related mortality, *excluding* STI-related mortality. This measure has previously been used to test PST-related hypotheses while reducing potential overlap with LHT-related interpretations (Brown et al., 2024). These analyses produced similar results, showing that higher pathogen mortality (absent STIs) predicts fewer interracial marriages, more racial preferences in online dating, and increased likelihood of preferring only one's same race (Tables S25, S26).

SI References

Akaliyski, P. (2024). Controlling for Non-Independence of Nations Should Not Be the Default Choice in Crosscultural Research: Response to Claessens et al. (2023) *(SSRN Scholarly Paper No. 5014193). Social Science Research Network.* https://doi.org/10.2139/ssrn.5014193

Al-Shawaf, L., Lewis, D. M. G., & Buss, D. M. (2017). Sex differences in disgust: Why are women more easily disgusted than men? *Emotion Review, 10(*2), 149–160. https://doi.org/10.1177/1754073917709940

Bouman, A., Heineman, M. J., & Faas, M. M. (2005). Sex hormones and the immune response in humans. *Human Reproduction Update, 11*(4), 411–423. https://doi.org/10.1093/humupd/dmi008

Brown, G. D. A., Walasek, L., Mullett, T. L., Quispe-Torreblanca, E. G., Fincher, C. L., Kosinski, M., & Stillwell, D. (2024). Political Attitudes and Disease Threat: Regional Pathogen Stress Is Associated With Conservative Ideology Only for Older Individuals. *Personality & Social Psychology Bulletin, 50*(11), 1581–1595. https://doi.org/10.1177/01461672231183199

Claessens, S., Kyritsis, T., & Atkinson, Q. D. (2023). Cross-national analyses require additional controls to account for the non-independence of nations. *Nature Communications, 14*(1), 5776. https://doi.org/10.1038/s41467-023-41486-1

DeBruine, L. M., Jones, B. C., Crawford, J. R., Welling, L. L. M., & Little, A. C. (2010). The health of a nation predicts their mate preferences: Cross-cultural variation in women's preferences for masculinized male faces. *Proceedings of the Royal Society B: Biological Sciences, 277*(1692), 2405–2410. https://doi.org/10.1098/rspb.2009.2184

Fincher, C. L., & Thornhill, R. (2012). Parasite-stress promotes in-group assortative sociality: The cases of strong family ties and heightened religiosity. *Behavioral and Brain Sciences, 35*(2), 61–79. https://doi.org/10.1017/S0140525X11000021

Gangestad, S. W., & Buss, D. M. (1993). Pathogen prevalence and human mate preferences. *Ethology and Sociobiology, 14*(2), 89–96. https://doi.org/10.1016/0162-3095(93)90009-7

Greenwald, A. G., Nosek, B. A., & Banaji, M. R. (2003). Understanding and using the implicit association test: I. An improved scoring algorithm. J*ournal of Personality and Social Psychology, 85*(2), 197–216. https://doi.org/10.1037/0022-3514.85.2.197

Hackman, J., & Hruschka, D. (2013). Fast life histories, not pathogens, account for state-level variation in homicide, child maltreatment, and family ties in the U.S. *Evolution and Human Behavior, 34*(2), 118–124. https://doi.org/10.1016/j.evolhumbehav.2012.11.002

Harrington, J. R., & Gelfand, M. J. (2014). Tightness–looseness across the 50 United States. *Proceedings of the National Academy of Sciences, 111*(22), 7990–7995. https://doi.org/10.1073/pnas.1317937111

Horita, Y., & Takezawa, M. (2018). Cultural Differences in Strength of Conformity Explained Through Pathogen Stress: *A Statistical Test Using Hierarchical Bayesian Estimation. Frontiers in Psychology, 9*. https://doi.org/10.3389/fpsyg.2018.01921

Imada, H., & Mifune, N. (2021). Pathogen Threat and In-group Cooperation. *Frontiers in Psychology, 12*, 678188. https://doi.org/10.3389/fpsyg.2021.678188

Kuppens, T., & Pollet, T. V. (2014). Mind the level: Problems with two recent nation-level analyses in psychology. *Frontiers in Psychology, 5.* https://doi.org/10.3389/fpsyg.2014.01110

Kusano, K., & Kemmelmeier, M. (2018). Ecology of Freedom: Competitive Tests of the Role of Pathogens, Climate, and Natural Disasters in the Development of Socio-Political Freedom. *Frontiers in Psychology, 9*. https://doi.org/10.3389/fpsyg.2018.00954

Kusano, K., & Kemmelmeier, M. (2020). Multi-level modelling of time-series cross-sectional data reveals the dynamic interaction between ecological threats and democratic development. *Royal Society Open Science, 7*(3), 191804. https://doi.org/10.1098/rsos.191804

Lee, A. J., & Zietsch, B. P. (2011). Experimental evidence that women's mate preferences are directly influenced by cues of pathogen prevalence and resource scarcity. *Biology Letters, 7*(6), 892–895. https://doi.org/10.1098/rsbl.2011.0454

Mathison, B. A., & Pritt, B. S. (2023). The Landscape of Parasitic Infections in the United States. *Modern Pathology, 36*(8), 100217. https://doi.org/10.1016/j.modpat.2023.100217

Moon, J. W., Krems, J. A., & Cohen, A. B. (2018). Religious People Are Trusted Because They Are Viewed as Slow Life-History Strategists. *Psychological Science, 29*(6), 947–960. https://doi.org/10.1177/0956797617753606

Moon, J. W., Krems, J. A., Cohen, A. B., & Kenrick, D. T. (2019). Is Nothing Sacred? Religion, Sex, and Reproductive Strategies. *Current Directions in Psychological Science, 28*(4), 361–365. https://doi.org/10.1177/0963721419838242

Murray, D. R., Trudeau, R., & Schaller, M. (2011). On the Origins of Cultural Differences in Conformity: Four Tests of the Pathogen Prevalence Hypothesis. *Personality and Social Psychology Bulletin, 37*(3), 318–329. https://doi.org/10.1177/0146167210394451

O'Shea, B. A., Watson, D. G., Brown, G. D. A., & Fincher, C. L. (2020). Infectious Disease Prevalence, Not Race Exposure, Predicts Both Implicit and Explicit Racial Prejudice Across the United States. *Social Psychological and Personality Science, 11*(3), 345–355. https://doi.org/10.1177/1948550619862319

Payne, B. K., & Hannay, J. W. (2021). Implicit bias reflects systemic racism. *Trends in Cognitive Sciences, 25*(11), 927–936. https://doi.org/10.1016/j.tics.2021.08.001

Payne, B. K., Vuletich, H.A., & Lundberg, K. B. (2017). The Bias of Crowds: How Implicit Bias Bridges Personal and Systemic Prejudice. *Psychological Inquiry, 28*(4), 233–248. https://doi.org/10.1080/1047840X.2017.1335568

Schmitt, D. P. (2005). Sociosexuality from Argentina to Zimbabwe: A 48-nation study of sex, culture, and strategies of human mating. *Behavioral and Brain Sciences, 28*(2), 247–275; discussion 275-311. https://doi.org/10.1017/s0140525x05000051

Thomson, R., Yuki, M., Talhelm, T., Schug, J., Kito, M., Ayanian, A. H., Becker, J. C., Becker, M., Chiu, C., Choi, H.-S., Ferreira, C. M., Fülöp, M., Gul, P., Houghton-Illera, A. M., Joasoo, M., Jong, J., Kavanagh, C. M., Khutkyy, D., Manzi, C., … Visserman, M. L. (2018). Relational mobility predicts social behaviors in 39 countries and is tied to historical farming and threat. *Proceedings of the National Academy of Sciences, 115*(29), 7521–7526. https://doi.org/10.1073/pnas.1713191115

Tingley, D., Yamamoto, T., Hirose, K., Keele, L., & Imai, K. (2014). mediation: R Package for Causal Mediation Analysis. *Journal of Statistical Software, 59*, 1–38. https://doi.org/10.18637/jss.v059.i05

Tybur, J. M., Bryan, A. D., Lieberman, D., Caldwell Hooper, A. E., & Merriman, L. A. (2011). Sex differences and sex similarities in disgust sensitivity. *Personality and Individual Differences, Special Issue on Personality and Economics, 51*(3), 343–348. https://doi.org/10.1016/j.paid.2011.04.003

Vandello, J. A., & Cohen, D. (1999). Patterns of individualism and collectivism across the United States. *Journal of Personality and Social Psychology, 77*(2), 279–292. https://doi.org/10.1037/0022-3514.77.2.279

Weaving, M., & Gelfand, M. J. (2024). Majority rule: Do minorities live in 'tighter' social worlds? *Current Opinion in Psychology, 60*, 101885. https://doi.org/10.1016/j.copsyc.2024.101885

Xu, K., Nosek, B., & Greenwald, A. (2014). Psychology data from the race implicit association test on the project implicit demo website. *Journal of Open Psychology Data, 2*(1). https://doi.org/10.5334/jopd.ac

Table S1.

Demographic information for online dating profiles in Study 2.

State	*Total N*	*Average Age in Years (sd)*	*% Female*	*% White*	*College Degree (% Associate or Greater)*
Alabama	352	33.2 (6.8)	50.9%	38.1%	47.7%
Alaska	216	34.1 (7.2)	25.0%	70.4%	44.0%
Arizona	340	34.6 (5.4)	50.9%	70.3%	74.7%
Arkansas	360	34.0 (6.3)	50.0%	61.4%	59.2%
California	328	34.7 (5.3)	51.2%	47.6%	70.7%
Colorado	349	34.7 (5.4)	50.4%	84.2%	77.4%
Connecticut	353	34.0 (5.9)	50.4%	69.4%	68.8%
Delaware	316	31.8 (6.9)	44.3%	64.2%	38.9%
Florida	360	32.7 (6.8)	51.1%	61.1%	60.6%
Georgia	352	34.3 (5.3)	51.1%	29.0%	73.6%
Hawaii	296	35.0 (5.6)	51.7%	27.7%	67.6%
Idaho	347	34.3 (6.1)	52.4%	92.8%	66.0%
Illinois	365	33.8 (6.4)	50.4%	82.2%	52.1%
Indiana	366	33.3 (6.2)	50.3%	79.2%	57.4%
Iowa	362	34.4 (6.0)	51.4%	87.3%	66.9%
Kansas	348	32.4 (7.0)	49.7%	76.7%	39.7%
Kentucky	273	33.1 (7.3)	34.4%	84.6%	34.4%
Louisiana	374	33.8 (6.4)	49.5%	61.8%	52.7%
Maine	266	32.7 (7.1)	31.6%	92.9%	35.0%
Maryland	361	33.4 (6.0)	49.9%	80.9%	68.7%
Massachusetts	338	33.8 (5.2)	53.3%	63.9%	79.6%
Michigan	356	33.4 (6.5)	49.7%	77.8%	67.7%
Minnesota	357	33.7 (5.5)	53.5%	79.6%	77.0%
Mississippi	356	32.7 (7.2)	50.3%	38.5%	49.2%
Missouri	342	32.0 (7.1)	46.5%	82.2%	41.8%
Montana	309	32.9 (6.8)	41.7%	93.9%	42.4%
Nebraska	368	33.2 (6.4)	49.2%	89.4%	67.1%
Nevada	259	33.1 (6.6)	30.9%	78.4%	32.4%
New Hampshire	315	32.0 (6.3)	42.2%	93.3%	49.5%
New Jersey	371	34.1 (5.9)	50.4%	73.3%	65.8%
New Mexico	236	34.1 (6.6)	30.5%	47.5%	48.3%
New York	341	34.1 (6.3)	51.3%	81.8%	73.9%
North Carolina	353	34.1 (5.8)	49.9%	61.8%	77.3%
North Dakota	358	33.0 (6.7)	49.4%	84.9%	52.5%
Ohio	363	33.6 (5.8)	50.1%	72.7%	57.9%
Oklahoma	341	33.6 (6.4)	48.7%	70.1%	50.7%
Oregon	354	33.8 (6.5)	49.4%	79.4%	48.3%
Pennsylvania	359	33.3 (6.1)	52.6%	77.4%	62.4%
Rhode Island	356	33.8 (5.8)	50.3%	79.5%	68.5%
South Carolina	358	33.9 (6.6)	51.4%	49.4%	64.2%
South Dakota	132	33.7 (6.9)	24.2%	78.8%	42.4%
Tennessee	361	33.8 (5.8)	50.4%	71.2%	72.6%
Texas	351	34.6 (5.8)	50.4%	55.0%	76.9%
Utah	355	34.4 (6.0)	52.1%	76.3%	62.3%
Vermont	275	33.9 (6.6)	33.1%	91.3%	48.7%
Virginia	370	34.3 (5.5)	50.8%	65.1%	71.4%
Washington	338	34.3 (6.7)	47.9%	82.0%	59.2%
West Virginia	367	33.4 (7.0)	49.0%	85.6%	45.5%
Wisconsin	357	34.3 (5.8)	51.3%	83.5%	76.2%
Wyoming	352	33.0 (6.6)	49.1%	83.2%	43.5%
**Total**	**16,732**	**33.7 (6.3)**	**48.1%**	**72.1%**	**59.5%**

Table S2.

Correlations among pathogen prevalence, interracial marriages, and interracial dating preferences.

Variable	1	2	3	4	5	6
1. Pathogen Prevalence						
2. Zoonotic Pathogens	.28*					
	[.00, .52]					
3. Non-zoonotic Pathogens	.91**	0.15				
	[.85, .95]	[-.14, .41]				
4. Interracial marriages (non-adjusted)	-0.02	-0.03	0.19			
	[-.30, .26]	[-.31, .25]	[-.09, .44]			
5. Interracial marriages (adjusted)	-.54**	-0.22	-.34*	.79**		
	[-.71, -.30]	[-.47, .06]	[-.56, -.06]	[.66, .88]		
6. Any racial preference	.66**	.39**	.60**	-0.01	-.45**	
	[.46, .79]	[.12, .60]	[.38, .75]	[-.29, .27]	[-.65, -.20]	
7. Exclusive same-race preference	.46**	.34*	.31*	-.40**	-.64**	.81**
	[.21, .66]	[.07, .57]	[.03, .54]	[-.61, -.14]	[-.78, -.44]	[.68, .89]

Note. M and *SD* are used to represent mean and standard deviation, respectively. Values in square brackets indicate the 95% confidence interval for each correlation. * $p < .05$. ** $p < .01$.

Table S3.

Regression results using state-level interracial and intraracial marriage rates as the criterion variable.

Model 1 examines the role of pathogen prevalence on interracial and intraracial marriages; Model 2 examines the role of state-level racial bias on interracial and intraracial marriages. Model 3 includes parameters for both pathogen prevalence and state-level racial bias.

A. Interracial marriages

	Model 1			Model 2			Model 3		
Predictors	*β*	*(95% CI)*	*p*	*β*	*(95% CI)*	*p*	*β*	*(95% CI)*	*p*
Pathogen Prevalence	-.75	-1.0 – -.50	<.001				-.58	-.88 – -.28	<.001
State-level racial bias				-.60	-.86 – -.34	<.001	-.27	-.56 – .01	.058
Anti-miscegenation laws	-.05	-.24 – .14	.614	.01	-.24 – .25	.948	.06	-.16 – .27	.602
Population density	-.28	-.43 – -.12	.001	-.05	-.25 – .16	.638	-.17	-.36 – .02	.082
Diversity Index	1.18	.98 – 1.39	<.001	.82	.64 – .99	<.001	1.11	.89 – 1.33	<.001
R^2 / R^2 adjusted	0.76 / 0.74			0.71 / 0.68			0.78 / 0.76		

B. Intra-racial marriages among Whites

	Model 1			Model 2			Model 3		
Predictors	*β*	*(95% CI)*	*p*	*β*	*(95% CI)*	*p*	*β*	*(95% CI)*	*p*
Pathogen Prevalence	.72	.51 – .92	<.001				.56	.31 – .80	<.001
State-level racial bias				.57	.35 – .79	<.001	.25	.02 – .49	.033
Anti-miscegenation laws	.03	-.13 – .19	.695	-.02	-.23 – .19	.854	-.07	-.24 – .11	.456
Population density	.23	.10 – .36	.001	.02	-.16 – .19	.862	.13	-.02 – .29	.097
Diversity Index	-1.25	-1.42 – -1.07	<.001	-.89	-1.04 – -.74	<.001	-1.18	-1.35 – -1.00	<.001
R^2 / R^2 adjusted	0.84 / 0.82			0.78 / 0.76			0.85 / 0.84		

C. Intra-racial marriages for all non-White racial/ethnic groups

	Model 1			Model 2			Model 3		
Predictors	*β*	*(95% CI)*	*p*	*β*	*(95% CI)*	*p*	*β*	*(95% CI)*	*p*
Pathogen Prevalence	.59	.30 – .87	<.001				.52	.16 – .88	.006
State-level racial bias				.40	.11 – .69	.008	.10	-.23 – .44	.537
Anti-miscegenation laws	.02	-.20 – .24	.874	.02	-.26 – .30	.879	-.02	-.60 –.50	.862
Population density	.23	.05 – .40	.015	.08	-.15 – .31	.509	.18	-.04 – .41	.109
Diversity Index	.23	-.01 – .47	.064	.52	.32 – .72	<.001	.25	-.00 – .51	.054
R^2 / R^2 adjusted	0.68 / 0.66			0.64 / 0.59			0.69 / 0.65		

Note. N=50 states. All coefficients are standardized. State-level racial bias represents the aggregated pro-White implicit bias task (IAT) scores. Anti-miscegenation laws represent a dummy variable of whether the state had anti-miscegenation laws at the point of the Loving vs. Virginia ruling (No = 0; Yes = 1).

Table S4.

Regression results for White daters using state-level dating preferences as the criterion variable. Models examine the role of pathogen prevalence on (A) the presence of a stated racial preference, (B) exclusive same-race preferences, and (C) exclusion of Black daters

**A**	Any racial preference								
	Model 1			Model 2			Model 2		
Predictors	*OR*	*(95% CI)*	*p*	*OR*	*(95% CI)*	*p*	*OR*	*(95% CI)*	*p*
**Fixed Effects**									
(Intercept)	0.23	(0.18 – 0.31)	***	0.22	(0.16 – 0.29)	***	0.24	(0.18 – 0.31)	***
Pathogen Prevalence	1.31	(1.19 – 1.43)	***				1.29	(1.17 – 1.42)	***
State-level racial bias				1.12	(1.01 – 1.24)	*	1.03	(0.94 – 1.12)	
Gender [Woman]	1.36	(1.25 – 1.49)	***	1.36	(1.25 – 1.48)	***	1.36	(1.25 – 1.49)	***
Age	1.01	(1.00 – 1.01)		1.01	(1.00 – 1.01)	†	1.01	(1.00 – 1.01)	
Education [College degree]	1.04	(0.96 – 1.14)		1.05	(0.96 – 1.15)		1.04	(0.96 – 1.14)	
Diversity Index (capital)	1.07	(0.98 – 1.16)		1.15	(1.04 – 1.27)	**	1.07	(0.99 – 1.17)	†
Anti-miscegenation laws	1.55	(1.28 – 1.88)	***	1.92	(1.52 – 2.41)	***	1.52	(1.25 – 1.86)	***
Capital population density	1.22	(1.13 – 1.32)	***	1.18	(1.06 – 1.32)	**	1.21	(1.11 – 1.31)	***
**Random Effects**									
$\sigma^2_{Residual}$ / $\tau_{00\ State}$ / ICC	3.29 / 0.04 / 0.01			3.29 / 0.08 / 0.02			3.29 / 0.04 / 0.01		
Marginal / Conditional R^2	0.064 / 0.076			0.057 / 0.079			0.064 / 0.076		

**B**	Exclusive same-race preference								
	Model 1			Model 2			Model 2		
	OR	*(95% CI)*	*p*	*OR*	*(95% CI)*	*p*	*OR*	*(95% CI)*	*p*
**Fixed Effects**									
(Intercept)	0.12	(0.08 – 0.16)	***	0.11	(0.08 – 0.16)	***	0.12	(0.08 – 0.17)	***
Pathogen Prevalence	1.25	(1.12 – 1.39)	***				1.18	(1.06 – 1.31)	**
State-level pro-White bias				1.23	(1.11 – 1.37)	***	1.16	(1.05 – 1.28)	**
Gender [Woman]	2.51	(2.23 – 2.82)	***	2.51	(2.23 – 2.81)	***	2.51	(2.23 – 2.82)	***
Age	0.99	(0.98 – 1.00)	*	0.99	(0.98 – 1.00)	*	0.99	(0.98 – 1.00)	*
Education [College degree]	1.06	(0.94 – 1.19)		1.06	(0.95 – 1.20)		1.06	(0.94 – 1.19)	
Diversity Index (capital)	0.94	(0.86 – 1.04)		1.01	(0.91 – 1.12)		0.97	(0.89 – 1.07)	
Anti-miscegenation laws	1.49	(1.18 – 1.88)	**	1.58	(1.26 – 1.99)	***	1.36	(1.09 – 1.70)	**
Capital population density	1.32	(1.20 – 1.45)	***	1.23	(1.11 – 1.37)	***	1.24	(1.13 – 1.37)	***
**Random Effects**									
$\sigma^2_{Residual}$ / $\tau_{00\ State}$ / ICC	3.29 / 0.05 / 0.02			3.29 / 0.06 / 0.02			3.29 / 0.04 / 0.01		
Marginal R^2 / Conditional R^2	0.105 / 0.118			0.108 / 0.124			0.109 / 0.120		

**C**	Excludes Black daters								
	Model 1			Model 2			Model 2		
	OR	*(95% CI)*	*p*	*OR*	*(95% CI)*	*p*	*OR*	*(95% CI)*	*p*
**Fixed Effects**									
(Intercept)	0.21	(0.16 – 0.28)	***	0.20	(0.15 – 0.26)	***	0.21	(0.16 – 0.28)	***
Pathogen Prevalence	1.31	(1.19 – 1.44)	***				1.29	(1.16 – 1.42)	***
State-level pro-White bias				1.13	(1.02 – 1.26)	*	1.04	(0.95 – 1.14)	
Gender [Woman]	1.30	(1.18 – 1.42)	***	1.29	(1.18 – 1.42)	***	1.30	(1.18 – 1.42)	***
Age	1.00	(0.99 – 1.01)		1.00	(0.99 – 1.01)		1.00	(0.99 – 1.01)	
Education [College degree]	1.09	(0.99 – 1.20)	†	1.10	(1.00 – 1.20)	†	1.09	(0.99 – 1.20)	†
Diversity Index (capital)	1.05	(0.96 – 1.14)		1.13	(1.02 – 1.25)	*	1.06	(0.97 – 1.15)	
Anti-miscegenation laws	1.24	(1.14 – 1.34)	***	1.20	(1.07 – 1.34)	**	1.22	(1.11 – 1.33)	***
Capital population density	1.70	(1.39 – 2.08)	***	2.08	(1.64 – 2.63)	***	1.66	(1.35 – 2.04)	***
**Random Effects**									
$\sigma^2_{Residual}$ / $\tau_{00\ State}$ / ICC	3.29 / 0.04 / 0.01			3.29 / 0.08 / 0.02			3.29 / 0.04 / 0.01		
Marginal R^2 / Conditional R^2	0.069 / 0.081			0.063 / 0.085			0.070 / 0.082		

Note. $^{\dagger}p$ <.10, **p* < .05, ***p* < .01, ****p* < .001

Table S5.
Coefficients for mediation models predicting state-level interracial marriages from state-level pathogen prevalence, as mediated by various state-level indices

Mediator	ACME	ADE	Total Effect	Percent Mediated
Tightness-Looseness				
All Interracial Marriages	-0.02	-0.74***	-0.76***	0.03
White Intra-racial Marriages	0.00	0.06***	0.06***	0.02
Non-White Intra-racial Marriages	0.15*	0.44**	0.59***	0.02*
Collectivism				
All Interracial Marriages	0.01	-0.76***	-0.76***	-0.00
White Intra-racial Marriages	0.00	0.06***	0.06***	0.00
Non-White Intra-racial Marriages	-0.00	0.59***	0.59***	0.00
Racial Bias				
All Interracial Marriages	-0.18*	-0.57***	-0.75***	0.23*
White Intra-racial Marriages	0.01*	0.05***	0.06***	0.22*
Non-White Intra-racial Marriages	0.06	0.52**	0.58***	0.11
Conformity				
All Interracial Marriages	0.00	-0.75***	-0.75***	-0.00
White Intra-racial Marriages	-0.00	0.06***	0.06***	-0.00
Non-White Intra-racial Marriages	-0.01	0.59**	0.59***	-0.01
Family Ties				
All Interracial Marriages	-0.01	-0.74***	-0.75***	0.00
White Intra-racial Marriages	0.00	0.06***	0.06***	0.00
Non-White Intra-racial Marriages	0.01	0.58***	0.59***	0.00

Note. Models include control variables. †p <.10, *p < .05, **p < .01, ***p < .001

Table S6.

Results of mediation models predicting individual-level racial preferences displayed on online dating profiles from state-level pathogen prevalence, as mediated by various state-level indices

	**Any Racial Preference**				**Same Race Preference**			
**Mediator**	ACME	ADE	Total Effect	Proportion Mediated	ACME	ADE	Total Effect	Proportion Mediated
Tightness-Looseness								
All Daters	0.0008	0.0409***	0.0416***	0.0184	0.0020†	0.0253***	0.0272***	0.0715†
White Daters	0.0032*	0.0549***	0.0581***	0.0559*	0.0028*	0.0282***	0.0315***	0.0905*
Non-White Daters	-0.0029	0.0094	0.0066	-0.2101	-0.0005	0.0136**	0.0130***	-0.0381
Collectivism								
All Daters	0.0092***	0.0314***	0.0405***	0.2230***	0.0069***	0.0196***	0.0264***	0.2613***
White Daters	0.0075**	0.0494***	0.0569***	0.1331**	0.0059**	0.0244***	0.0303***	0.1962**
Non-White Daters	0.0134**	-0.0068	0.0065	1.1149	0.0079***	0.0047	0.0129**	0.6136**
Racial Bias								
All Daters	0.0001	0.0413***	0.0413***	0.0042	0.0050**	0.0224***	0.0274***	0.1832**
White Daters	0.0016	0.0560***	0.0576***	0.0263	0.0072***	0.0248***	0.0320***	0.2269***
Non-White Daters	-0.0031	0.0091	0.0060	-0.1992	-0.0006	0.0135**	0.0126*	-0.0468
Conformity								
All Daters	-0.0001	0.0418***	0.0413***	-0.0148	-0.0005	0.0278***	0.0273***	-0.0176
White Daters	-0.0005	0.0580***	0.0576***	-0.0060	-0.0005	0.0319***	0.0314***	-0.0159
Non-White Daters	-0.0014	0.0081	0.0067	-0.1044	-0.0002	0.0129**	0.0127**	-0.0160
Family Ties								
All Daters	0.0068***	0.0340***	0.0409***	0.169***	0.0027†	0.0245***	0.0272***	0.0976†
White Daters	0.0054**	0.0516***	0.0569***	0.0943**	0.0014	0.0297***	0.0311***	0.0457
Non-White Daters	0.0112**	-0.0053	0.0060	0.9313	0.0046*	0.0082	0.0128**	0.3645*

Note. Models include control variables. †p <.10, *p < .05, **p < .01, ***p < .001

Table S7.

Results of mediation models examining pathogen prevalence as a mediator of the effect of state-level racial bias (state-level IAT scores) on racial preferences displayed on online dating profiles

**Any Racial Preference**				
	ACME	ADE	Total Effect	Proportion Mediated
All Daters	0.0129***	-0.0001	0.0128**	1.0009**
White Daters	0.0156***	0.0032	0.0188***	0.8461***
Non-White Daters	0.0033	-0.0084	-0.0050	-0.2560
**Same Race Preference**				
	ACME	ADE	Total Effect	Proportion Mediated
All Daters	0.0071***	0.0122***	0.0192***	0.3676***
White Daters	0.0070***	0.0166***	0.0236***	0.2998***
Non-White Daters	0.0047*	-0.0013	0.0034	0.5887
**Exclusion of Black Daters by White Daters**				
	ACME	ADE	Total Effect	Proportion Mediated
White Daters	0.0145***	0.0049	0.0194***	0.7459***

Note. Models include control variables. †p <.10, *p < .05, **p < .01, ***p < .001

Table S8

Results of Bayesian Spatial Gaussian regression models accounting for longitude and latitude, examining impact of Pathogen Prevalence on Interracial and Intra-racial Marriages (Study 1)

A. Interracial Marriages

	Estimate	Error	95% CI	Rhat	Bulk ESS	Tail ESS
Intercept	0.00	0.26	-0.57 – 0.33	1.01	319	77
Pathogen Prevalence	-0.76	0.13	-1.02 – -0.49	1	2555	2921
Diversity Index (DI)	1.19	0.12	0.96 – 1.41	1	2956	2376
Anti-Miscegenation laws	-0.10	0.22	-0.53 – 0.31	1	3209	1232
Population Density	-0.27	0.09	-0.45 – -0.09	1	3480	2366
Gaussian Process Hyperparameters:						
sdgp(gplatlong)	0.24	0.28	0.01 – 1.03	1.01	328	112
lscale(gplatlong)	0.27	1.48	0.01 – 1.78	1.02	284	138
Further Distributional Parameters:						
sigma	0.5	0.08	0.32 – 0.64	1	376	212

B. Intra-racial Marriages (White couples)

	Estimate	Error	95% CI	Rhat	Bulk ESS	Tail ESS
Intercept	-0.02	0.15	-0.27 – 0.24	1.01	1376	1082
Pathogen Prevalence	0.69	0.11	0.47 – 0.91	1	2100	2175
Diversity Index (DI)	-1.22	0.1	-1.42 – -1.02	1	2175	2162
Anti-Miscegenation laws	0.07	0.18	-0.28 – 0.41	1	2857	3064
Population Density	0.21	0.08	0.05 – 0.36	1	2051	2170
Gaussian Process Hyperparameters:						
sdgp(gplatlong)	0.24	0.2	0.01 – 0.62	1.01	528	993
lscale(gplatlong)	0.11	0.35	0.01 – 0.70	1.03	138	706
Further Distributional Parameters:						
sigma	0.39	0.07	0.23 – 0.52	1.02	240	276

C. Intra-racial Marriages (non-White same-race couples)

	Estimate	Error	95% CI	u-95%	CI	Rhat
Intercept	-0.02	0.32	-0.63 – 0.70	1.01	654	216
Pathogen Prevalence	0.58	0.15	0.28 – 0.87	1	2981	3117
Diversity Index (DI)	0.23	0.13	-0.03 – 0.49	1	2898	2922
Anti-Miscegenation laws	0.04	0.25	-0.44 – 0.54	1	2895	2630
Population Density	0.23	0.11	0.01 – 0.44	1	3341	2568
Gaussian Process Hyperparameters:						
sdgp(gplatlong)	0.33	0.39	0.01 – 1.46	1	534	274
lscale(gplatlong)	0.23	0.79	0.01 – 1.71	1.02	309	219
Further Distributional Parameters:						
Sigma	0.57	0.08	0.40 – 0.73	1.01	528	220

Table S9

Results of Bayesian Spatial Gaussian regression models accounting for longitude and latitude (Study 2)

A. Exclusive same-race preference

	Est.	Error	95% CI	Rhat	Bulk ESS	Tail ESS
Intercept	-3.38	0.54	-4.47 – -2.09	1.01	1561	1214
Pathogen Prevalence	0.21	0.06	0.10 – 0.32	1	1766	2220
Race (White)	1.07	0.07	0.94 – 1.20	1	4000	2691
Gender (Woman)	0.95	0.05	0.84 – 1.06	1	5560	3141
Age	-0.01	0	-0.02 – 0.00	1	5083	3224
College degree	0.06	0.05	-0.04 – 0.17	1	4495	2525
Diversity Index	-0.03	0.05	-0.12 – 0.08	1.01	1028	2110
Anti-miscegenation laws	0.31	0.12	0.07 – 0.55	1	1644	2089
Population density	0.20	0.06	0.09 – 0.31	1.01	912	1863
Gaussian Process Hyperparameters:						
sdgp	0.95	0.98	0.02 – 3.86	1.01	296	542
lscale	1.1	1.58	0.02 – 4.53	1.02	209	90
Multilevel Hyperparameters:						
sd(Intercept)	0.18	0.04	0.12 – 0.26	1.01	762	507

B. Any racial preference

	Est.	Error	95% CI	Rhat	Bulk ESS	Tail ESS
Intercept	-2.00	0.89	-3.97 – 0.04	1	1158	946
Pathogen Prevalence	0.17	0.05	0.08 – 0.26	1	1243	1920
Race (White)	0.17	0.04	0.09 – 0.26	1	4804	1454
Gender (Woman)	0.39	0.04	0.32 – 0.46	1	4981	2894
Age	0.01	0.00	0.01 – 0.02	1	6709	3068
College degree	0.09	0.04	0.02 – 0.16	1	4373	2388
Diversity Index	0.08	0.04	0 – 0.15	1	1782	2204
Anti-miscegenation laws	0.30	0.10	0.11 – 0.50	1.01	1021	2355
Population density	0.19	0.04	0.11 – 0.28	1	1525	2207
Gaussian Process Hyperparameters:						
Sdgp	0.64	0.66	0.04 – 2.66	1.01	443	337
Lscale	0.64	4.24	0.02 – 2.44	1.01	361	366
Multilevel Hyperparameters:						
sd(Intercept)	0.22	0.05	0.12 – 0.32	1	940	747

C. Exclusive same race preference (White daters only)

	Est.	Error	95% CI	Rhat	Bulk ESS	Tail ESS
Intercept	-3.92	0.59	-4.97 – -2.60	1.06	59	33
Pathogen Prevalence	0.20	0.1	0.01 – 0.38	1.01	1153	2985
Gender (Woman)	1.11	0.14	0.85 – 1.39	1.02	235	2308
Age	0.01	0.01	-0.01 – 0.03	1.02	261	2610
College degree	0.14	0.12	-0.11 – 0.37	1.01	453	1626
Diversity Index	-0.05	0.07	-0.18 – 0.09	1	2512	2730
Anti-miscegenation laws	0.32	0.21	-0.08 – 0.74	1.02	402	2927
Population density	0.07	0.09	-0.11 – 0.25	1.02	2034	2268
Gaussian Process Hyperparameters:						
sdgp	0.26	0.26	0.01 – 1.01	1.02	385	612
lscale	0.29	0.69	0.01 – 1.72	1.02	245	614
Multilevel Hyperparameters:						
sd(Intercept)	0.24	0.06	0.12 – 0.35	1	815	673

D. Exclusive same-race preference (non-White daters only)

	Est.	Error	95% CI	Rhat	Bulk ESS	Tail ESS
Intercept	-2.18	0.31	-2.76 – -1.50	1.01	1049	436
Pathogen Prevalence	0.21	0.06	0.09 – 0.34	1	1394	2035
Gender (Woman)	0.92	0.06	0.81 – 1.04	1	2790	2518
Age	-0.01	0.00	-0.02 – 0	1	4115	2875
College degree	0.05	0.06	-0.06 – 0.17	1	3588	2600
Diversity Index	-0.04	0.06	-0.16 – 0.06	1	1202	2201
Anti-miscegenation laws	0.37	0.14	0.1 – 0.64	1	1106	1535
Population density	0.24	0.06	0.13 – 0.36	1	1325	2216
Gaussian Process Hyperparameters:						
sdgp	0.57	0.71	0.02 – 2.97	1.06	85	32
lscale	0.82	2.27	0.02 – 9.59	1.06	64	19
Multilevel Hyperparameters:						
sd(Intercept)	0.15	0.1	0.01 – 0.35	1.01	957	1767

Table S10

Results of Study 1 examining the effect of non-Zoonotic and Zoonotic pathogens on interracial and intra-racial marriages

**Non-Zoonotic Infections**	Interracial Marriages	Intra-racial marriages White	Intra-racial marriages non-White
Predictors	*β*	*β*	*β*
Non-Zoonotic Disease	-.68***	.61***	.53**
Diversity Index (2010)	1.25***	.12	-1.23***
Anti-miscegenation laws	-.16	.08	.18†
Population Density	-.41***	.34***	.34***
R^2 / R^2 adjusted	.69 / .66	.67 / .63	.73 / .71

**Zoonotic Infections**			
Predictors	*β*	*β*	*β*
Zoonotic Disease	-.04	.12	.15
Diversity Index (2010)	.77***	-.86***	.54***
Anti-miscegenation laws	-.40***	.35***	.27*
Population Density	-.28*	.20†	.17
R^2 / R^2 adjusted	.56 / .52	.66 / .63	.58 / .54

Note. †p <.10, *p < .05, **p < .01, ***p < .001

Table S11.

Effect of non-Zoonotic and Zoonotic disease on stated racial preferences (Study 2)

A. Non-Zoonotic Diseases

	All daters		White daters		Non-White daters	
	Any Racial Preference	Same Race Preference	Any Racial Preference	Same Race Preference	Any Racial Preference	Same Race Preference
Predictors	*OR p*	*OR p*	*OR p*	*OR p*	*OR p*	*OR p*
(Intercept)	0.16***	0.03***	0.23***	0.11***	0.10***	0.02***
Non-Zoonotic Disease	1.22***	1.19**	1.25***	1.16*	1.15**	1.22*
Race (White = 1)	1.18***	2.90***				
Gender (Woman = 1)	1.48***	2.58***	1.36***	2.50***	1.90***	3.10***
Age	1.01***	0.99†	1.01	0.99*	1.02***	1.01
Education	1.09*	1.06	1.04	1.06	1.22**	1.14
Capital Diversity Index	1.06	0.94	1.05	0.94	0.99	0.92
Capital Population Density	1.26***	1.31***	1.26***	1.35***	1.23†	1.50*
Loving Repeal	1.53***	1.63***	1.71***	1.68***	1.24***	1.10
Random Effects						
σ^2	3.29	3.29	3.29	3.29	3.29	3.29
τ_{00}	0.03 $_{State}$	0.06 $_{State}$	0.05 $_{State}$	0.07 $_{State}$	0.03 $_{State}$	0.01 $_{State}$
ICC	0.01	0.02	0.02	0.02	0.01	0
Observations	16732 / 50		12061 / 50		4671 / 50	
Marginal R^2 / Conditional R^2	0.06 / 0.07	0.14 / 0.16	0.06 / 0.08	0.10 / 0.12	0.06 / 0.07	0.12 / 0.13

B. Zoonotic Diseases

	All daters		White daters		Non-White daters	
	Any Racial Preference	Same Race Preference	Any Racial Preference	Same Race Preference	Any Racial Preference	Same Race Preference
Predictors	*OR p*	*OR p*	*OR p*	*OR p*	*OR p*	*OR p*
(Intercept)	0.15***	0.03***	0.21***	0.11***	0.10***	0.02***
Zoonotic Disease	1.07	1.11†	1.10†	1.10	0.99	1.08
Race (White = 1)	1.18***	2.87***				
Gender (Woman = 1)	1.48***	2.58***	1.36***	2.51***	1.91***	3.10***
Age	1.01***	0.99†	1.01†	0.99*	1.02***	1.01
Education	1.09*	1.07	1.05	1.06	1.23**	1.14
Capital Diversity Index	1.12**	0.99	1.13*	0.99	1.00	0.91
Capital Population Density	1.20***	1.24***	1.18**	1.28***	1.45***	1.80***
Loving Repeal	1.83***	1.86***	2.06***	1.89***	1.22***	1.04
Random Effects						
σ^2	3.29	3.29	3.29	3.29	3.29	3.29
τ_{00}	0.06 $_{State}$	0.08 $_{State}$	0.08 $_{State}$	0.08 $_{State}$	0.04 $_{State}$	0.02 $_{State}$
ICC	0.02	0.02	0.02	0.02	0.01	0
Observations / States	16732 / 50		12061 / 50		4671 / 50	
Marginal R^2 / Conditional R^2	0.05 / 0.07	0.15 / 0.16	0.06 / 0.08	0.10 / 0.12	0.05 / 0.06	0.12 / 0.12

Note. †p <.10, *p < .05, **p < .01, ***p < .001, σ^2 =3.29

Table S12.
Examination of Gender × Pathogen interaction effects for the presence of any racial preference (left) and exclusive same-race preferences (right)

	Any Racial Preference			Exclusive same-race preference		
Predictors	OR	95% CI	*p*	OR	95% CI	*p*
(Intercept)	0.16	0.12 – 0.20	<.001	0.04	0.03 – 0.05	<.001
Pathogen Prevalence	1.28	1.18 – 1.40	<.001	1.22	1.08 – 1.37	.001
Race (White = 1)	1.18	1.09 – 1.28	<.001	2.92	2.56 – 3.35	<.001
Gender (Woman = 1)	1.50	1.39 – 1.62	<.001	2.56	2.30 – 2.85	<.001
Age	1.01	1.01 – 1.02	<.001	0.99	0.98 – 1.00	.06
Education	1.10	1.02 – 1.18	.015	1.07	0.96 – 1.18	.237
Capital Diversity Index	1.08	1.00 – 1.16	.036	0.95	0.87 – 1.03	.225
Capital Population Density	1.22	1.14 – 1.31	<.001	1.28	1.17 – 1.39	<.001
	1.47	1.24 – 1.75	<.001	1.45	1.17 – 1.80	.001
Anti-Miscegenation laws						
Gender × Pathogen Prevalence	0.90	0.84 – 0.97	.003	1.06	0.96 – 1.17	.276
Random Effects						
σ^2	3.29			3.29		
τ_{00}	0.03 State			0.05 State		
ICC	0.01			0.01		
Observations	16732 / 50			16732 /50		
Marginal R^2 / Conditional R^2	0.057 / 0.067			0.140 / 0.152		

Table S13.
Results of Study 1 examining the effect of teenage pregnancy rate on interracial and intra-racial marriages

	Interracial Marriages	Intra-racial marriages White	Intra-racial marriages non-White
Predictors	*β*	*β*	*β*
Teenage Pregnancy Rate	.01	-.01	.02
Diversity Index (2010)	.76***	-.84***	.53***
Anti-miscegenation laws	-.90**	.85**	.51†
Population Density	-.29*	.23*	.19*
R^2 / R^2 adjusted	.56 / .52	.66 / .62	.57 / .53

Note. †*p* <.10, **p* < .05, ***p* < .01, ****p* < .001

Table S14.

Examination of the effect of Teenage Pregnancy Rate on the presence of any racial preference (left) and exclusive same-race preferences (right)

	Any Racial Preference			Exclusive same-race preference		
Predictors	OR	95% CI	*p*	OR	95% CI	*p*
(Intercept)	0.14	0.09 – 0.20	<.001	0.04	0.02 – 0.06	<.001
Teenage Pregnancy Rate	1.00	0.99 – 1.02	.691	0.99	0.97 – 1.02	.631
Race (White = 1)	1.17	1.08 – 1.27	<.001	2.85	2.50 – 3.26	<.001
Gender (Woman = 1)	1.48	1.37 – 1.59	<.001	2.58	2.32 – 2.87	<.001
Age	1.01	1.01 – 1.02	<.001	0.99	0.98 – 1.00	.066
Education	1.10	1.02 – 1.18	.016	1.07	0.96 – 1.19	.210
Capital Diversity Index	1.12	1.03 – 1.22	.008	0.99	0.89 – 1.10	.802
Capital Population Density	1.25	1.13 – 1.38	<.001	1.28	1.13 – 1.45	<.001
Anti-Miscegenation laws	1.84	1.48 – 2.28	<.001	2.05	1.56 – 2.70	<.001
Random Effects						
σ^2	3.29			3.29		
τ_{00}	0.06 $_{State}$			0.09 $_{State}$		
ICC	0.02			0.03		
Observations	16732 / 50			16732 / 50		
Marginal R^2 / Conditional R^2	0.049 / 0.066			0.138 / 0.160		

Table S15.

Results of Study 1 examining the effect of age at first birth on interracial and intra-racial marriages

	Interracial Marriages	Intra-racial marriages	
		White	non-White
Predictors	*β*	*B*	*β*
Age at First Birth	.03	-.01	-.14
Diversity Index (2010)	.76***	-.85***	.57***
Anti-miscegenation laws	-.83**	.79**	.48†
Population Density	-.32*	.26*	.32*
R^2 / R^2 adjusted	.56 / .52	.65 / .62	.57 / .53

Note. †*p* <.10, **p* < .05, ***p* < .01, ****p* < .001

Table S16.

Examination of the effect of Age at First Birth on the presence of any racial preference (left) and exclusive same-race preferences (right)

	Any Racial Preference			Exclusive same-race preference		
Predictors	OR	95% CI	*p*	OR	95% CI	*p*
(Intercept)	0.12	0.09 – 3.05	.201	0.03	0.00 – 1.51	.078
Age at first birth	1.01	0.90 – 1.12	.923	1.01	0.88 – 1.15	.928
Race (White = 1)	1.17	1.08 – 1.27	<.001	2.86	2.50 – 3.27	<.001
Gender (Woman = 1)	1.48	1.37 – 1.59	<.001	2.58	2.32 – 2.87	<.001
Age	1.01	1.01 – 1.02	<.001	0.99	0.98 – 1.00	.066
Education	1.09	1.02 – 1.18	.017	1.07	0.96 – 1.19	.207
Capital Diversity Index	1.12	1.03 – 1.22	.008	0.98	0.88 – 1.09	.764
Capital Population Density	1.23	1.11 – 1.37	<.001	1.30	1.14 – 1.47	<.001
Anti-Miscegenation laws	1.90	1.55 – 2.31	<.001	1.98	1.54 – 2.54	<.001
Random Effects						
σ^2	3.29			3.29		
τ_{00}	0.06 State			0.09 State		
ICC	0.02			0.03		
Observations	16732 / 50			16732 / 50		
Marginal R^2 / Conditional R^2	0.049 / 0.066			0.137 / 0.160		

Table S17.

Results of Study 1 examining the effect of teenage pregnancy rate and pathogen prevalence on interracial and intra-racial marriages

	Interracial Marriages	Intra-racial marriages White	Intra-racial marriages non-White
Predictors	*β*	*B*	*β*
Pathogen Prevalence	-.79***	.76	.59***
Teenage Pregnancy Rate	.03	-.03*	-.00
Diversity Index (2010)	1.17***	-1.24***	.23
Anti-miscegenation laws	-.27	.12	.04
Population Density	-.20*	.15†	.22*
R^2 / R^2 adjusted	.78 / .75	.85 / .84	.68 / .65

Note. †*p* <.10, **p* < .05, ***p* < .01, ****p* < .001

Table S18.

Examination of the effect of Teenage Pregnancy Rate and Pathogen Prevalence on the presence of any racial preference (left) and exclusive same-race preferences (right)

	Any Racial Preference			Exclusive same-race preference		
Predictors	OR	95% CI	*p*	OR	95% CI	*p*
(Intercept)	0.17	0.12 – 0.24	<.001	0.05	0.03 – 0.07	<.001
Pathogen Prevalence	1.22	1.13 – 1.33	<.001	1.28	1.16 – 1.42	<.001
Teenage Pregnancy Rate	1.00	0.98 – 1.01	.590	0.99	0.97 – 1.00	.123
Race (White = 1)	1.18	1.09 – 1.28	<.001	2.91	2.55 – 3.33	<.001
Gender (Woman = 1)	1.48	1.37 – 1.59	<.001	2.58	2.32 – 2.87	<.001
Age	1.01	1.01 – 1.02	<.001	0.99	0.98 – 1.00	.062
Education	1.09	1.02 – 1.18	.018	1.06	0.96 – 1.18	.255
Capital Diversity Index	1.08	1.01 – 1.16	.029	0.95	0.87 – 1.03	.227
Capital Population Density	1.50	1.24 – 1.82	<.001	1.58	1.25 – 2.00	<.001
Anti-Miscegenation laws	1.21	1.12 – 1.31	<.001	1.23	1.12 – 1.36	<.001
Random Effects						
σ^2	3.29			3.29		
τ_{00}	0.03 State			0.04 State		
ICC	0.01			0.01		
Observations	16732 / 50			16732 /50		
Marginal R^2 / Conditional R^2	0.055 / 0.065			0.142 / 0.153		

Table S19.

Results of Study 1 examining the effect of age at first birth and pathogen prevalence on interracial and intra-racial marriages

	Interracial Marriages	Intra-racial marriages White	Intra-racial marriages non-White
Predictors	β	β	β
Pathogen Prevalence	-.81***	.79***	.59***
Age at First Birth	-.18	.20*	.01
Diversity Index (2010)	1.24***	-1.31***	.22†
Anti-miscegenation laws	-.23	.10	.05
Population Density	-.18	.13	.10
R^2 / R^2 adjusted	.78 / .75	.85 / .84	.68 / .65

Note. †p <.10, *p < .05, **p < .01, ***p < .001

Table S20.

Examination of the effect of Age at First Birth and Pathogen Prevalence on the presence of any racial preference (left) and exclusive same-race preferences (right)

	Any Racial Preference			Exclusive same-race preference		
Predictors	OR	95% CI	*p*	OR	95% CI	*p*
(Intercept)	0.09	0.01 – 1.26	.074	0.02	0.00 – 0.54	.02
Pathogen Prevalence	1.22	1.12 – 1.32	<.001	1.26	1.14 – 1.39	<.001
Age at First Birth	1.02	0.93 – 1.11	.698	1.02	0.91 – 1.14	.741
Race (White = 1)	1.18	1.09 – 1.28	<.001	2.91	2.55 – 3.33	<.001
Gender (Woman = 1)	1.48	1.37 – 1.59	<.001	2.58	2.32 – 2.87	<.001
Age	1.01	1.01 – 1.02	<.001	0.99	0.98 – 1.00	.061
Education	1.09	1.02 – 1.18	.017	1.07	0.96 – 1.18	.235
Capital Diversity Index	1.08	1.00 – 1.16	.036	0.95	0.87 – 1.03	.204
Capital Population Density	1.49	1.24 – 1.80	<.001	1.48	1.17 – 1.86	.001
Anti-Miscegenation laws	1.21	1.12 – 1.32	<.001	1.27	1.14 – 1.40	<.001
Random Effects						
σ^2	3.29			3.29		
τ_{00}	0.03 $_{State}$			0.05 $_{State}$		
ICC	0.01			0.01		
Observations	16732 / 50			16732 /50		
Marginal R^2 / Conditional R^2	0.055 / 0.064			0.141 / 0.153		

Table S21.
Examination of the effect of Pathogen Prevalence on the total number of racial categories excluded from online dating preferences

	Number of Races Excluded		
Predictors	β	95% CI	*p*
(Intercept)	0.64	0.38 – 0.90	<.001
Pathogen Prevalence	0.22	0.13 – 0.31	<.001
Race (White = 1)	0.31	0.22 – 0.41	<.001
Gender (Woman = 1)	0.77	0.69 – 0.86	<.001
Age	0.00	-0.00 – 0.01	.333
Education	0.04	-0.04 – 0.13	.329
Capital Diversity Index	0.03	-0.05 – 0.11	.443
Capital Population Density	0.23	0.15 – 0.31	<.001
Anti-Miscegenation laws	0.42	0.22 – 0.61	<.001
Random Effects			
σ^2	6.83		
τ_{00}	0.04 $_{State}$		
ICC	0.01		
Observations	16732 / 50		
Marginal R^2 / Conditional R^2	0.044 / 0.050		

Table S22.
Examination of the effect of Pathogen Prevalence on the exclusion of more than one racial category from online dating preferences

	Two or more races excluded		
Predictors	OR	95% CI	*p*
(Intercept)	0.14	0.11 – 0.18	<.001
Pathogen Prevalence	1.22	1.13 – 1.32	<.001
Race (White = 1)	1.22	1.12 – 1.33	<.001
Gender (Woman = 1)	1.83	1.69 – 1.97	<.001
Age	1.01	1.00 – 1.01	.04
Education	1.03	0.95 – 1.11	.477
Capital Diversity Index	1.06	0.99 – 1.14	.073
Capital Population Density	1.22	1.14 – 1.31	<.001
Anti-Miscegenation laws	1.44	1.22 – 1.70	<.001
Random Effects			
σ^2	3.29		
τ_{00}	0.03 $_{State}$		
ICC	0.01		
Observations	16732 / 50		
Marginal R^2 / Conditional R^2	0.065 / 0.073		

Table S23.

Examination of the effect of Pathogen Prevalence on the exclusion of specific individual racial categories from online dating preferences

	Racial Category Excluded from Preferences:							
	White	Black	Hispanic	Asian	Native American	Pacific Islander	Middle Eastern	East Indian
Predictors	OR	OR	OR	OR	OR	OR	OR	OR
(Intercept)	0.04***	0.08***	0.07***	0.08***	0.13***	0.13***	0.13***	0.14***
Pathogen Prevalence	1.14†	1.17**	1.19**	1.20***	1.22***	1.21***	1.23***	1.22***
Race (White = 1)	0.11***	2.49***	1.62***	1.39***	1.22***	1.27***	1.30***	1.29***
Gender (Woman = 1)	2.02***	1.27***	2.56***	2.70***	1.99***	1.90***	1.90***	1.83***
Age	1.01	1.00	1.00	1.00	1.00	1.00	1.01	1.01
Education	1.00	1.14**	1.03	1.00	1.07	0.99	1.01	1.01
Capital Diversity Index	0.96***	1.16***	0.99	0.98	1.03	1.02	1.06	1.07
Capital Population Density	1.17*	1.18**	1.25***	1.28***	1.24***	1.24***	1.21***	1.22***
Anti-Miscegenation laws	1.72	1.40***	1.47**	1.57***	1.33**	1.48***	1.42***	1.43***
R^2 / R^2 adjusted	0.29 / 0.30	0.07 / 0.09	0.10 / 0.12	0.11 / 0.12	0.07 / 0.08	0.07 / 0.08	0.07 / 0.08	0.07 / 0.07

Note. †*p* <.10, *p < .05, ***p* < .01, ****p* < .001

Table S24.

Examination of the effect of Pathogen Prevalence on the exclusion of specific individual racial categories from online dating preferences by daters who are not of the same race

	Racial Category Excluded from Preferences:							
	White	Black	Hispanic	Asian	Native American	Pacific Islander	Middle Eastern	East Indian
Predictors	OR	OR	OR	OR	OR	OR	OR	OR
(Intercept)	0.04***	0.17***	0.11***	0.11***	0.15***	0.16***	0.15***	0.16***
Pathogen Prevalence	1.22*	1.28***	1.14***	1.17***	1.20***	1.18***	1.20***	1.19***
Gender (Woman = 1)	2.19***	1.36***	2.46***	2.67***	2.00***	1.89***	1.88***	1.81***
Age	1.01	1.00	1.00	1.00	1.00	1.00	1.01*	1.01*
Education	1.22*	1.17***	1.04	1.04	1.08	1.01	1.04	1.04
Capital Diversity Index	0.93	1.06	1.00	1.00	1.02	1.02	1.05	1.06
Capital Population Density	1.19**	1.22***	1.23***	1.27***	1.23***	1.23***	1.21***	1.22***
Anti-Miscegenation laws	1.58**	1.63***	1.40**	1.51***	1.31**	1.45***	1.40***	1.42***
R^2 / R^2 adjusted	0.09 / 0.10	0.06 / 0.08	0.08 / 0.10	0.10 / 0.11	0.07 / 0.08	0.06 / 0.07	0.06 / 0.07	0.06 / 0.07

Note. †p <.10, *p < .05, **p < .01, ***p < .001

Table S25.

Results of Study 1 examining the effect of pathogen mortality (without STI-related mortality) on interracial and intra-racial marriages

	Interracial Marriages	Intra-racial marriages	
		White	non-White
Predictors	*β*	*β*	*β*
Pathogen Mortality	-.20*	.01	.18*
Diversity Index (2010)	.83***	-.07***	.49***
Anti-miscegenation laws	-.34	.04*	.16
Population Density	-.28**	.25**	.23*
R^2 / R^2 adjusted	.62 / .59	.67 / .64	.61 / .58

Note. †*p* <.10, **p* < .05, ***p* < .01, ****p* < .001

Table S26.

Examination of the effect of pathogen mortality (without STI-related mortality) on the presence of any racial preference (left) and exclusive same-race preferences (right)

	Any Racial Preference			Exclusive same-race preference		
Predictors	OR	*95% CI*	*p*	OR	95% CI	*p*
(Intercept)	0.09	0.05 – 0.13	<.001	0.02	0.01 – 0.03	<.001
Pathogen Mortality	1.09	1.02 – 1.15	.005	1.10	1.02 – 1.18	.009
Race (White = 1)	1.18	1.09 – 1.28	<.001	2.87	2.51– 3.29	<.001
Gender (Woman = 1)	1.48	1.37 – 1.59	<.001	2.58	2.32 – 2.87	<.001
Age	1.01	1.01 – 1.02	<.001	0.99	0.98 – 1.00	.062
Education	1.10	1.02 – 1.18	.016	1.07	0.96 – 1.19	.207
Capital Diversity Index	1.12	1.04 – 1.21	.004	0.99	0.89– 1.09	.766
Capital Population Density	1.21	1.11 – 1.31	<.001	1.26	1.14– 1.40	<.001
Anti-Miscegenation laws	1.49	1.18 – 1.87	.001	1.48	1.11 – 1.98	.008
Random Effects						
σ^2	3.29			3.29		
τ_{00}	0.05 State			0.07 State		
ICC	0.01			0.02		
Observations	16732 / 50			16732 /50		
Marginal R^2 / Conditional R^2	0.052 / 0.066			0.139 / 0.157		

Figure S1

Heat maps of prevalence of racial preferences (left) and exclusive same race preferences (right) across all U.S. states

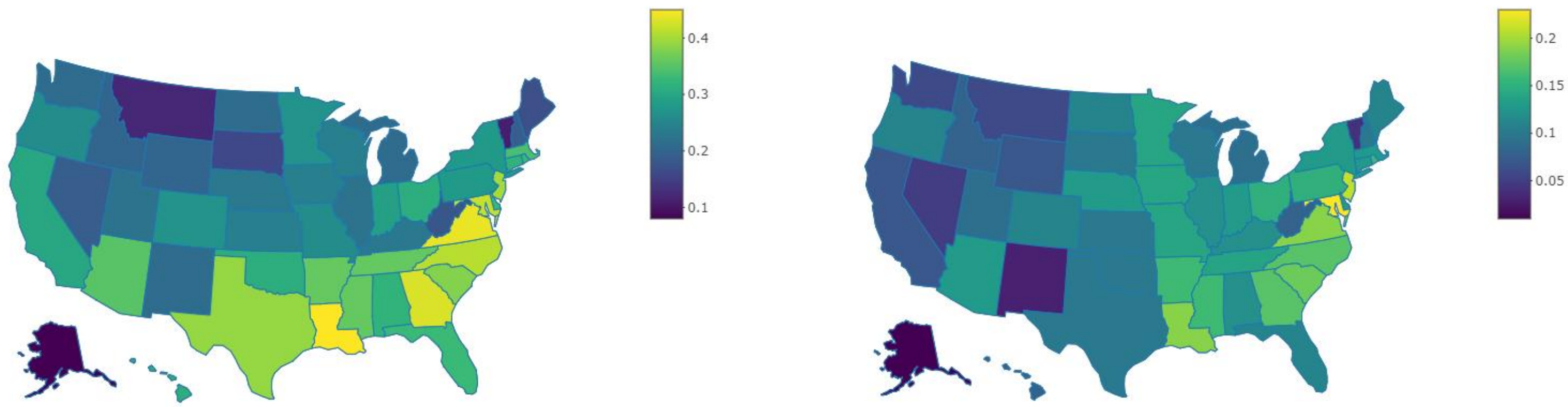

Figure S2

Heat maps of prevalence of intermarriage (left) and historical pathogen prevalence (right) across all U.S. states

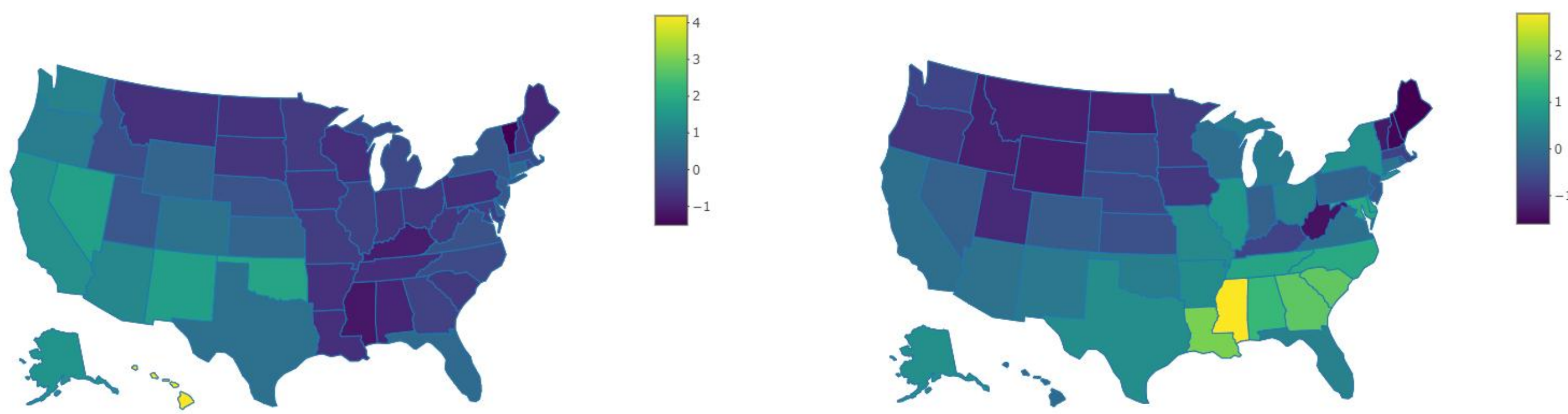

Figure S3

Visualization of interaction between gender and pathogens on the presence of any racial preference. Error bands represent the 95% CI.

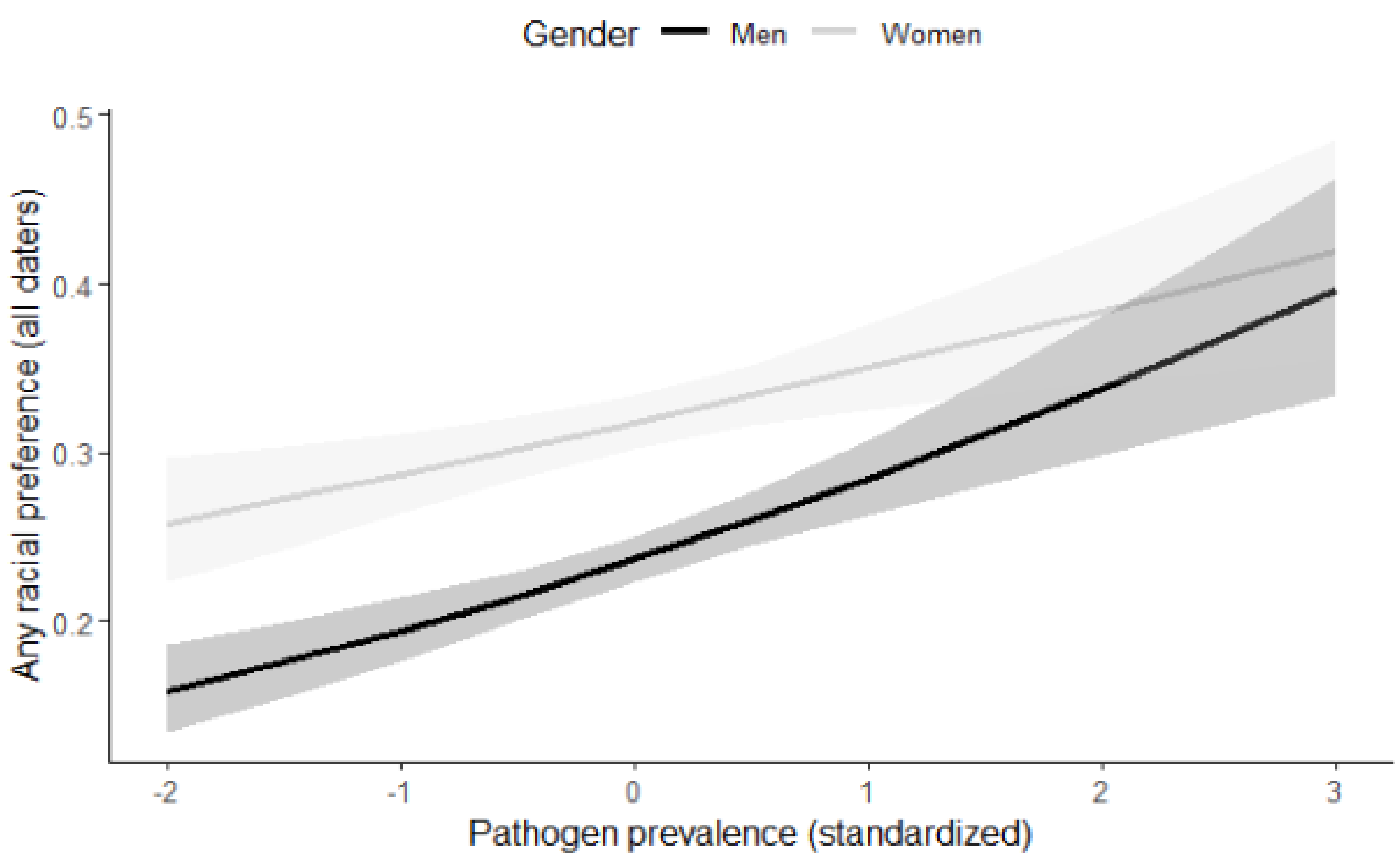

Supplement: Supplementary file 1 [file pgag356_supplementary_data.pdf]
